# Servo Navigation and Phase Equalization Enhanced by Run‐Time Stabilization (PEERS) for 3D EPI Time Series

**DOI:** 10.1002/mrm.70555

**Published:** 2026-08-30

**Authors:** Malte Riedel, Thomas Ulrich, Samuel Bianchi, Klaas P. Pruessmann

**Affiliations:** ^1^ Institute for Biomedical Engineering ETH Zurich and University of Zurich Zurich Switzerland

**Keywords:** EPI, feedback control, field correction, head motion, motion correction, orbital navigators

## Abstract

**Purpose:**

To enhance time‐resolved segmented imaging by synergy of run‐time stabilization and retrospective data‐driven phase correction.

**Methods:**

A segmented 3D EPI sequence for fMRI time series is equipped with servo navigation based on short orbital navigators and a linear perturbation model, enabling run‐time correction for rigid‐body motion, bulk phase, and frequency fluctuations. Complementary retrospective phase correction is based on the repetitive structure of the time series and serves to address residual phase and frequency offsets. The combined approach is termed phase equalization enhanced by run‐time stabilization (PEERS).

**Results:**

The proposed methods are assessed in terms of resulting alignment and tSNR of 3D EPI time series in a phantom and in vivo. Servo navigation is found to diminish motion confound in raw data and maintain k‐space consistency over time series. Coarse frequency tracking based on short navigators is supplemented by precise shot‐wise frequency and phase correction using retrospective phase equalization of EPI data. Relative to conventional volume realignment, PEERS achieved tSNR improvements of up to 30% for small motion and on the order of 10% when volunteers tried to hold still.

**Conclusion:**

Servo navigation achieves high‐precision run‐time motion correction for 3D EPI and is synergistic with retrospective phase equalization. Fully automatic and self‐calibrated, PEERS offers effective plug‐and‐play motion and phase correction for stabilized 3D EPI time series. The translation of stability gains into metrics of functional sensitivity is complex and must be studied with suitable statistical power. The data obtained in this work does not support any conclusions in this regard.

## 1 Introduction

Functional magnetic resonance imaging (fMRI) requires fast volume acquisitions to resolve BOLD signal variations [1, 2, 3] in space and time. Due to its fast sampling and inherent robustness, two‐dimensional echo‐planar imaging (2D EPI) [4] has become a standard technique for fMRI acquisitions. 3D EPI [5, 6] offers intrinsically higher sensitivity and more benign spin history‐related motion artifacts than 2D EPI, but makes volume acquisition more vulnerable to physiological noise [7, 8]. Head motion and magnetic field fluctuations over the course of 3D scans contaminate image time series and statistical data analysis, posing further challenges for fMRI with 3D EPI [9, 10, 11, 12, 13, 14, 15, 16].

In principle, head motion can be addressed by robust sampling, motion prevention, and motion correction [17, 18, 19]. Rapidly sampled volume acquisitions in fMRI are in fact fairly robust to motion, yet inter‐volume motion and artifacts due to intra‐volume motion can still persist [8]. Motion prevention techniques like dedicated patient preparation [20], bite bars [21], cushioning or sedation [22] can be used to reduce motion artifacts with varying costs and effectiveness. In addition, a wide range of prospective and retrospective techniques have been proposed to effectively correct head motion in various scan settings based on external sensor hardware [14, 23, 24, 25, 26, 27], MR‐based navigators [28, 29, 30, 31, 32, 33, 34, 35, 36, 37], or data‐driven self‐navigation [38, 39, 40, 41, 42, 43].

Navigator‐based motion corrections offer high‐precision motion tracking without the need for external hardware, but at the cost of sequence interference [18]. Recently, methods using short navigators of a few milliseconds have been proposed [35, 37, 44], which can be easily inserted into standard fMRI sequences. The servo navigation method [37, 44] uses short navigator readouts (≈3 ms) and a self‐calibrating linear model for run‐time motion and frequency correction. Once implemented, such navigated sequences offer autonomous, easy‐to‐use operation, making motion correction more accessible for a wide range of studies.

In addition to head motion, magnetic field fluctuations are another confounding factor in image time series [45, 46, 47]. There are many causes for field variations, including breathing [48, 49], pose‐dependent field changes [18, 50, 51], scanner drift or sampling‐related eddy currents [52, 53], which have different properties regarding spatial complexity and time evolution. Again, sensing mechanisms include external hardware [54, 55, 56, 57, 58, 59, 60], MR navigators [36, 37, 44, 61, 62, 63], and data‐driven techniques [62], which can be used in reconstruction or at run time.

Scans with repeated volume acquisitions as in fMRI additionally offer the opportunity to leverage redundancies along the time series not only for volume alignment [15, 43] but also for phase correction [64, 65, 66, 67, 68, 69, 70]. The *dynamic off‐resonance in k‐space* (DORK) method [68] provides shot‐wise phase and frequency corrections (temporal first‐order phase model) from a two‐point phase fit between image raw data in the k‐space center and a navigator echo. In contrast, self‐navigation techniques monitor shot‐to‐shot changes in phase accrual from image raw data alone. Shot‐wise phase correction of zeroth‐order in time was originally demonstrated for 2D FLASH imaging [66] and has recently been extended to first‐order phase correction in 3D EPI for MR thermometry in phantoms and tissue samples [70]. All of these techniques rely on stable k‐space consistency over time, and are thus sensitive to head rotations.

The goal of the present work is to improve the robustness of 3D EPI times series by synergy of run‐time stabilization, using servo navigation, and retrospective, data‐driven phase correction as proposed by Parker et al. [70]. Servo navigation with motion and frequency update [37] is included in a segmented 3D EPI sequence. The navigator's frequency precision proves to be critical for robust navigator‐based EPI phase correction, and dedicated correction strategies are investigated. Self‐navigated retrospective correction [70] fine‐tunes per‐shot phase and frequency estimates, exploiting redundancy due to repetition along the time series. The combined approach, phase equalization enhanced by run‐time stabilization (PEERS), is assessed in terms of alignment and tSNR in a phantom and in vivo, with and without motion. Special attention is given to the synergy between the two strategies in comparison with their individual performance.

## 2 Methods

### 2.1 Servo Navigation for Segmented 3D EPI

The concept of servo navigation has previously been studied for 3D gradient echo imaging [37, 44] and is, in this work, integrated into a segmented 3D EPI sequence. The sequence diagram is shown in Figure 1A and consists of a slab‐selective excitation (Ex) and an EPI readout. In each EPI shot, the sequence blips through the phase encoding dimension (P), while varying prephasers and rephasers in the slice encoding dimension (S). In this way, one k‐space plane per shot is acquired with repetition time $T_{R}$, yielding a full volume after $N_{z}$ slice encodings with volume acquisition time $T_{vol}=T_{R}\cdot N_{z}$.

**FIGURE 1:**
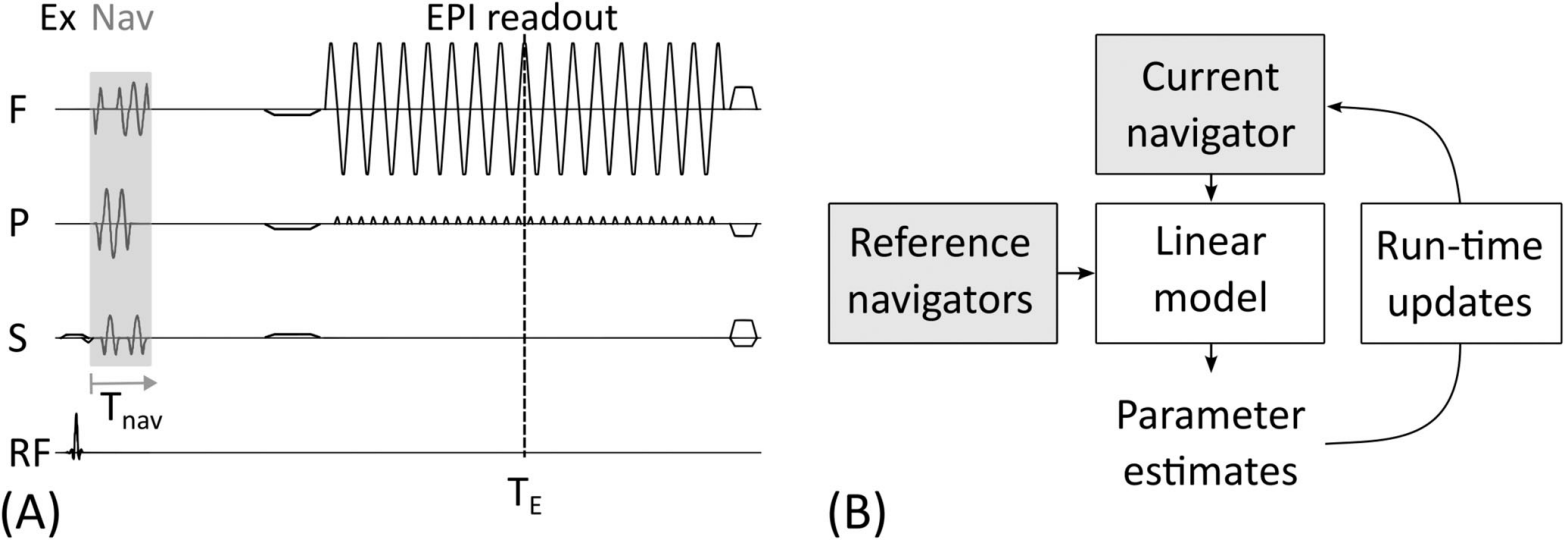
Servo navigation for segmented 3D EPI. (A) Sequence diagram. (B) Servo navigation overview. A 3D orbital navigator (Nav) with adaptable duration $T_{nav}$ is inserted between excitation (Ex) and EPI readout. After steady‐state build‐up, reference navigators are acquired on the fly to calibrate the linear model for motion and field parameter estimation. The parameters are used for run‐time scan geometry control closing the feedback loop for updated linear parameter estimation.

Servo navigation [37, 44] relies on three basic components (Figure 1B): a navigator, a model for parameter estimation, and a mechanism for run‐time scan control. First, a 3D orbital navigator [37], acquiring orbits around three orthogonal axes sequentially, at a k‐space radius of 400 rad/m, is inserted between excitation and the echo‐planar readout as shown in Figure 1A. The duration of the navigator $T_{nav}$ can be varied and is, by default, set to 3.2 ms. Second, a linear model of signal change as a function of motion, field, and phase parameters is determined on the fly, from four reference navigators acquired in four subsequent TRs [37]. The model matrix M [37] relates the parameter vector $\vec{\Delta }$ to change in navigator signal:

(1) $$\vec{s}\left(\vec{\Delta }\right)-{\vec{s}}^{\;(0)}=M\vec{\Delta },$$

where the vectors $\vec{s}\left(\vec{\Delta }\right)$ and ${\vec{s}}^{\;(0)}$ contain the complex‐valued signals of the current navigator and a reference navigator and $\vec{\Delta }$ includes six 3D rigid motion parameters, a global phase Δϕ and a global frequency parameter Δω. ${\vec{s}}^{\;(0)}$ is acquired as a separate (fifth) reference navigator to decorrelate noise between the matrix columns and the navigators [44]. Third, run‐time updates counter motion and field changes by rotating gradients and shifting slab‐selective excitation. This process forms a control loop that stabilizes both the imaging process and the navigator acquisition itself [31], keeping the signal model valid beyond the initial linear range.

### 2.2 Navigator‐Based Phase Correction

Relative parameter updates of global phase Δϕ and frequency Δω are accumulated over the scan as described in [37], yielding absolute time courses for the total global phase $\Delta \phi_{total,j}$ and frequency $\Delta \omega_{total,j}$, with shot index j. Phase correction of image raw data is then achieved by the phase shift:

(2) $$\Delta \varphi_{j}(t)=\Delta \omega_{corr,j}\;t+\Delta \phi_{corr,j},$$

where t is the intra‐shot sampling time. $\Delta \omega_{corr,j}$ and $\Delta \phi_{corr,j}$ are the navigator traces for the total global phase and frequency after optional filtering over time:

(3) $$\begin{array}{ll} \Delta \phi_{corr,j} & ={\text{filter}}_{j}(\Delta \phi_{total,j}), \end{array}$$

(4) $$\begin{array}{ll} \Delta \omega_{corr,j} & ={\text{filter}}_{j}(\Delta \omega_{total,j}). \end{array}$$

The phase correction in Equation (2) is, however, prone to noise amplification for long echo times or sampling durations, because the noise in the frequency parameter $\Delta \omega_{corr,j}$ is amplified linearly with the acquisition time t. Requirements for field parameter precisions, therefore, depend on the sequence timing. Filtering (Equations (3) and (4)) serves to reduce noise amplification, at the expense of effective controller bandwidth (ability to correct high‐frequency changes). Alternatively, the navigator acquisition time $T_{nav}$ can be made longer to increase field sensitivity and SNR at the expense of time and flexibility.

As an alternative strategy, phase correction with relative timing can be used to suppress noise amplification for short volume acquisitions:

(5) $$\Delta \varphi_{\text{rel},j}(t)=\Delta \varphi_{j}(t-T_{E})=\Delta \omega_{corr,j}\;\cdot \;(t-T_{E})+\Delta \phi_{corr,j}.$$

Here, phase correction is performed with timing $t_{rel}=(t-T_{E})$ relative to echo time $T_{E}$ and neglects the shot‐wise phase $\Delta \omega_{corr,j}\;T_{E}$ within a volume acquisition. By this, the phase along the k‐space line ${\left(0,0,k_{z}\right)}^{T}$ remains unchanged. Relative timing is beneficial when the frequency variation of $\Delta \omega_{corr,j}$ within a volume acquisition is dominated by noise rather than actual field evolution, which in our experience was typically the case for fast fMRI volume acquisitions of a few seconds at 3 T.

### 2.3 PEERS for EPI Trajectories

fMRI scans possess a strongly repetitive structure with minor local BOLD‐induced contrast variations. The redundancy along the EPI time series can be leveraged to perform self‐navigated frequency and phase corrections per shot relative to a peer shot from the reference volume as proposed by Parker et al. [70]. Head rotations, however, violate the k‐space correspondences between the shot peers and can potentially render the method ineffective or even harmful. We propose PEERS, combining the phase equalization by Parker et al. [70] with run‐time motion correction.

Algorithm 1 describes the retrospective processing similar to [70]. Each volume repetition with index d consists of $N_{l}$ EPI segments with a given trajectory, and each segment l is composed of $N_{m}$ echoes, forming the echo train. Each repetition‐segment pair (d, l) is associated with a running shot index $j=l+(d-1)N_{l}$ over the whole scan. An EPI shot obtains a 3D data array $S_{d,l}$ of shape $N_{m}\times N_{k}\times N_{c}$, where m, k, and c are the echo, readout sample, and coil indices. $T_{l}$ is a $N_{m}\times N_{k}\times 1$ array comprising the EPI readout timing. A shot‐wise global phase $\Delta \phi_{\text{epi},j}$ and frequency $\Delta \omega_{\text{epi},j}$ is estimated for each shot from the phase difference vector $\Delta {\vec{\varphi }}_{d,l}$ ($N_{m}\times 1$) to its peer. An overview of the PEERS method is shown in Figure 2. The method without run‐time motion correction is simply termed *phase equalization*.

**FIGURE 2:**
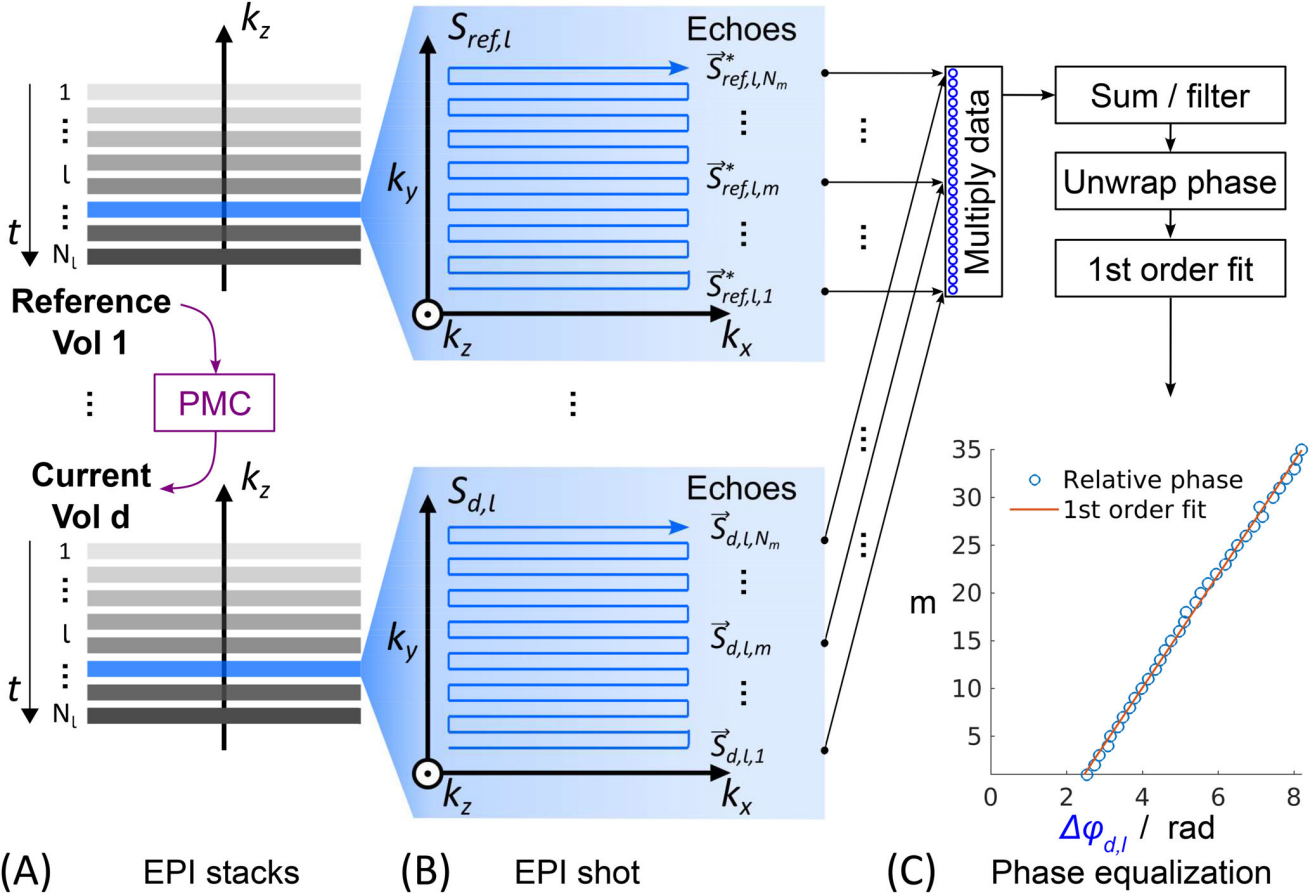
Phase equalization enhanced by run‐time stabilization (PEERS). (A) Shots from the first EPI stack (volume) serve as a reference for phase equalization of subsequent shots after prospective motion correction (PMC). (B) For any subsequent shot from volume d, relative phases are calculated echo‐by‐echo by scalar products of each echo signal ${\vec{S}}_{d,l,m}$ with its respective reference peer. m and l are echo and slice encoding indices. (C) Phases are unwrapped along the EPI echo train (m) followed by a first‐order fit yielding frequency (slope) and phase (offset) parameters.

ALGORITHM 1 **Self‐navigated phase equalization for segmented 3D EPI based on [70]. ∘, , and ∡ are element‐wise product, complex conjugation, and phase extraction.**

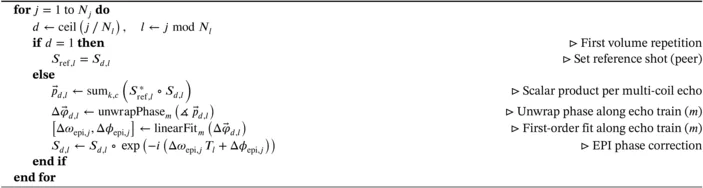

This algorithm corrects shot phase variations to the first temporal order with respect to its peer and, by this, matches the frequency‐ and phase‐induced point spread function to that of the reference volume, leading to stabilized voxel time series. The reference volume can be chosen arbitrarily in the reconstruction if it is corrupted. Unaware of the causes of phase variations, the correction includes both system‐ and physiology‐related effects like field changes, RF phase drifts, and breathing [70]. The sensitivity of the EPI sampling to frequency variations naturally adapts to variations in echo time, sampling duration, and field strength. The technique can generally handle arbitrary EPI phase encoding schemes and segmentations as long as they are repeated for each volume.

### 2.4 Phase Equalization for Arbitrary Trajectories

Phase equalization based on [70] is tailored to EPI trajectories with separable echoes. An extension of Algorithm 1 to arbitrary trajectories is possible by collapsing the echo (m) and intra‐echo readout (k) dimensions of the EPI signal array $S_{d,l}$ ($N_{m}\times N_{k}\times N_{c}$) into a collapsed readout ($k^{\prime }$) dimension ($N_{k^{\prime }}\times N_{c}$). The EPI‐specific implementation uses the noise‐robust complex‐valued sum over the echo‐wise readout dimension, which represents an inherent magnitude‐weighting for the phase estimation as described in [70]. For arbitrary trajectories, the complex‐valued sum can be replaced by mean filtering along the collapsed readout dimension ($k^{\prime }$) sorted in time, yielding the complex‐valued vector ${\vec{p}}_{d,l}\leftarrow {\text{meanFilter}}_{k^{\prime }}\left({\text{sum}}_{c}\left(S_{\text{ref},l}^{\;*}\;\circ \;S_{d,l}\right)\right)$ of length $N_{k^{\prime }}$. Finally, the time array $T_{l}$ needs to be adapted to the collapsed readout dimension with shape $N_{k^{\prime }}\times 1$ for the final phase correction.

### 2.5 Acquisition Details

The scan protocol consisted of three 3D EPI runs lasting 10 min each: (i) without servo navigation, (ii) with servo navigation ($T_{nav}=3.2\;\mathrm{ms}$), and (iii) with servo navigation and a long navigator ($T_{nav}=11.5\;\mathrm{ms}$). For case (iii), the navigator trajectory was stretched in time while keeping the same number of samples to increase the SNR. The order of the three runs was randomized and unknown to the subject. The scan parameters were: 2.5mm isotropic resolution, FOV = $230\times 200\times 140\;mm^{3}$ (incl. 1.4‐fold slice oversampling), $N_{z}=32$ slice encoding steps with linear ordering, reduction factors 2.2×1.75 (phase × slice), flip angle = 17.2°, TE = 30ms, $T_{R}$ = 64ms, $T_{vol}$ = 2.0s, and 300 volumes after 10 initial dummy volumes to build up the steady state. SPIR fat suppression [71] was used and its spoiler gradients were kept static in the initial orientation, because occasionally servo navigation instabilities were observed that probably stem from mechanical vibrations. A T1‐weighted MP‐RAGE scan [72] was acquired at isotropic resolution 1.1mm for anatomical reference.

The method was evaluated in a phantom and in vivo. For the phantom experiments, a sphere filled with small PMMA balls and a copper sulfate solution was used as described in [37]. In vivo, the scan sets were performed in six healthy volunteers in accordance with local ethical regulations on a 3T Philips (Best, The Netherlands) Ingenia scanner with a 32‐channel head coil. To approach the experimental conditions of fMRI scans, a visuomotor task was performed with a fully randomized event‐related design showing images of arrows presented in the upper or lower visual field. Subjects were asked to perform left‐ or right‐handed button presses according to the direction of the displayed arrows. Respiratory and cardiac physiology was monitored using a breathing belt and a pulse oximeter.

To study the influence of motion, 3‐min scans (100 volumes) with and without servo navigation were added for two of the six subjects with instructed motion. Subjects repeated a succession of rotations starting from a reference position and changing pose every 10s to [0, +1°(p), 0, −1°(p), 0, +1°(y), 0, −1°(y)] with p and y being pitch (nod) and yaw (head shake) rotations.

### 2.6 Reconstruction Details

The image reconstruction was implemented in MATLAB R2022b (MathWorks Inc., Natick, MA, USA) using the ReconFrame pipeline (Gyrotools, Zurich, Switzerland) with several modifications. After some basic vendor‐specific corrections, the k‐space data was corrected for the total global phase and frequency parameters of the navigators' run‐time estimates with relative timing (Equation (5)). A non‐causal median filter of size 9 (∼0.6s) was used for parameter filtering. After gridding of the EPI ramp samples, the k‐space data was corrected for the shifts in readout and phase directions according to the navigators' run‐time shift estimates. This was done in the reconstruction because only the slab shift was treated in run‐time by the scanner.

Next, EPI‐based phase equalization was optionally performed, estimating shot‐wise phases and frequencies by self‐navigation as described in Algorithm 1. A Nyquist ghosting correction was calculated from a vendor‐provided pre‐scan and applied in hybrid space followed by a ReconFrame implementation of regularized SENSE [73] in image space. All navigator‐based corrections were turned off if servo navigation was disabled. Sensitivity maps were reconstructed from the vendor's reference scan in the beginning of the scan set and included masking and smoothing.

Motion traces were pre‐processed differently for *Servo off* and *Servo on* to obtain total subject motion. For *Servo off*, shifts in phase encoding direction (RL) from volume realignment contain both subject motion and EPI shifting from frequency drift. To approximately separate and correct the frequency drift per volume in Servo off*, phase changes $\varphi_{d}=∡({\vec{V}}_{0}^{H}{\vec{V}}_{d})$ of the dth reconstructed volume ${\vec{V}}_{d}$ to the reference volume ${\vec{V}}_{0}$ were calculated, unwrapped along time ($\varphi_{d}^{\prime }$), interpreted as frequency $f_{0,d}=\varphi_{d}^{\prime }/(2\pi T_{E})$ and used to correct RL shifts for EPI shifting:

(6) $$\mathrm{EPI}\_\mathrm{shift}=f_{0,d}\;\;\text{ETS}\;\;{\text{FOV}}_{RL}$$

with the echo time shift ETS. For *Servo on*, frequency drifts are corrected by the navigator. To obtain total motion per volume, the rigid transforms from run‐time servo navigation (mean per volume) and retrospective volume realignment were multiplied. Frame‐to‐frame motion was quantified by framewise displacement (FD) [74] and absolute motion excursion by mean‐absolute displacement (MD), which is defined here analog to FD by: $\text{MD}=|\bar{x}|+|\bar{y}|+|\bar{z}|+50\;\text{mm}\cdot (|\bar{\alpha }|+|\bar{\beta }|+|\bar{\gamma }|)$ with mean shifts $\left[\bar{x},\bar{y},\bar{z}\right]$ and rotations $\left[\bar{\alpha },\bar{\beta },\bar{\gamma }\right]$ over time.

Statistical analysis was performed using SPM12 [75], including volume realignment [76], structural image co‐registration, and tissue segmentation into gray and white matter (WM), CSF, skull, soft tissue, and background [77]. Physiological traces from the respiration belt and pulse oximeter were processed using PhysIO [78]. tSNR was calculated before and after SPM volume realignment as the voxel‐wise mean over time divided by the voxel‐wise standard deviation (std) over time: ${\text{tSNR}}_{r}={\text{mean}}_{d}(\rho_{r,d})/{\text{std}}_{d}(\rho_{r,d})$, where $\rho_{r,d}$ is the magnitude signal of a voxel at location r and volume (time) index d.

## 3 Results

### 3.1 Run‐Time Motion Correction Example

Figure 3 shows an in vivo example with instructed motion for *Servo off* and *Servo on* comparing mean and standard deviation (std) of the voxel time series as well as the realignment parameters. For *Servo off*, the anterior part of the mean volume appears blurred, while the standard deviation shows strongly enhanced edges and choppy patterns throughout the brain in the logarithmic image. In contrast, the blurring is mitigated by *Servo on*, and the imprint of anatomical features and choppy patterns in the standard deviation images is clearly reduced. Edge enhancement mainly originates from frequency drift‐induced EPI shifting that is corrected by servo navigation, whereas choppy patterns are caused by motion‐ and phase‐induced shot inconsistencies.

**FIGURE 3:**
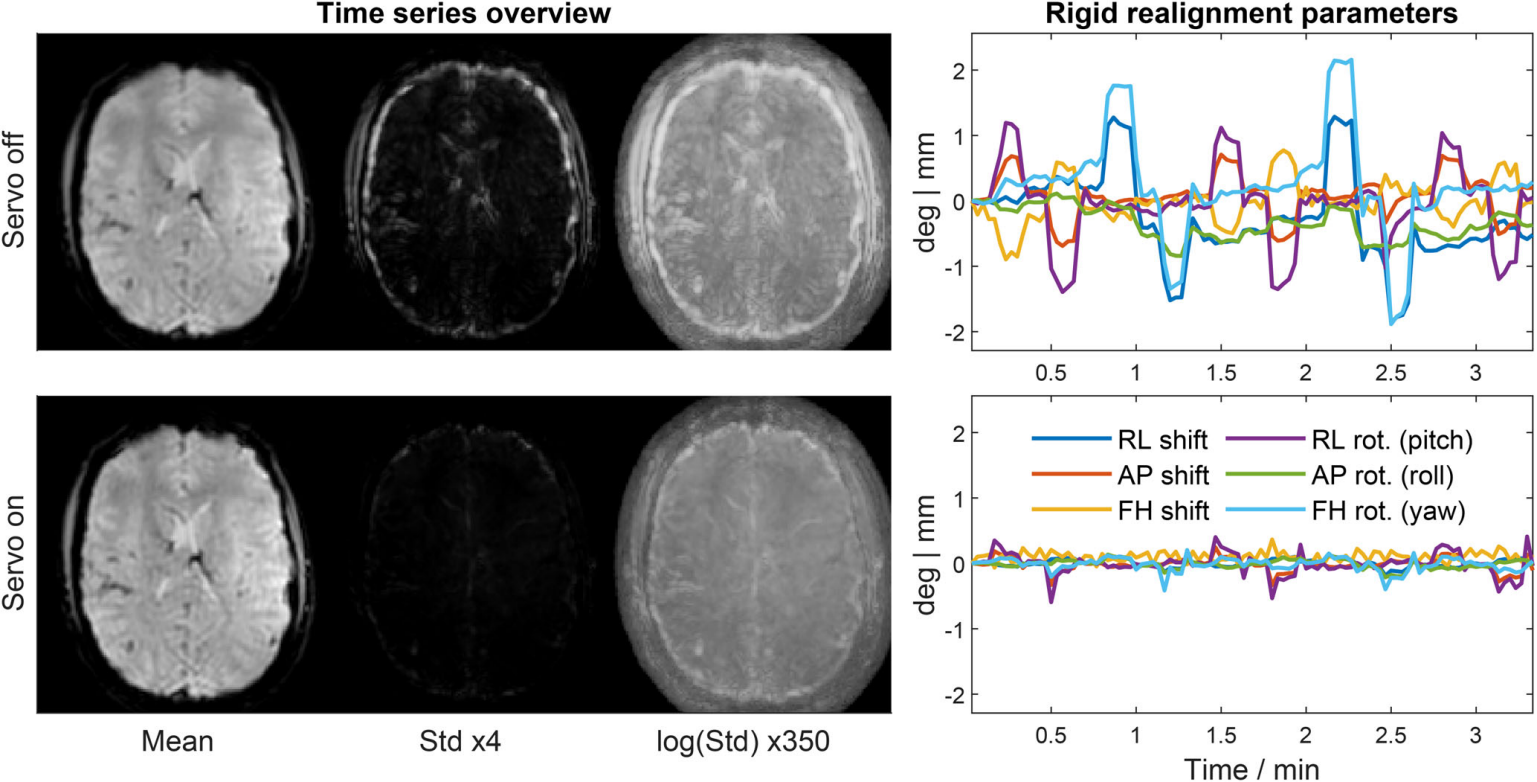
In vivo example with instructed motion comparing *Servo off* and *Servo on*. (Left): Mean and standard deviation (Std) of the voxel time series. (Right): Realignment parameters. For *Servo off*, the mean image is blurred and the std shows strong edge enhancement and choppy patterns in the logarithmic standard deviation, which are clearly mitigated by *Servo on*. The realignment parameters for *Servo off* show the instructed motion pattern, while being close to zero for *Servo on* due to effective run‐time motion control.

The volume realignment parameters for *Servo off* show the instructed motion pattern of successive pitch and yaw rotations, which are left uncorrected in the data. For *Servo on*, the volume realignment detects only minor motions close to zero that are left after the run‐time motion correction. However, the onset of the motion events is still visible with *Servo on* by small spikes and plateaus, especially for the pitch rotation (purple).

Figure S1 shows an equivalent in vivo example without instructed motion. A reconstruction example with a volume‐wise $f_{0}$ correction as described for Equation (6) is shown by Servo off* and separates the effect of EPI shifting from motion and intra‐volume frequency variations.

### 3.2 Realignment Parameter Statistics

Figure 4 shows the volume realignment parameter statistics and RMSE results for the data from all subjects without instructed motion for Servo off* (black boxes) and *Servo on* (green boxes). RMSE, median realignment parameters, and inter‐quartile ranges are consistently reduced by servo navigation for all parameters. For *Servo off*, shifts in EPI phase encoding direction (RL) include both subject motion and EPI shifting from frequency drifts. Volume realignment is incapable of differentiating between the two if no frequency stabilization is applied, resulting in large variations (gray box). For Servo off*, frequency‐induced EPI shifts are retrospectively corrected. For *Servo on*, the navigator corrects for both subject motion and frequency variations in run‐time.

**FIGURE 4:**
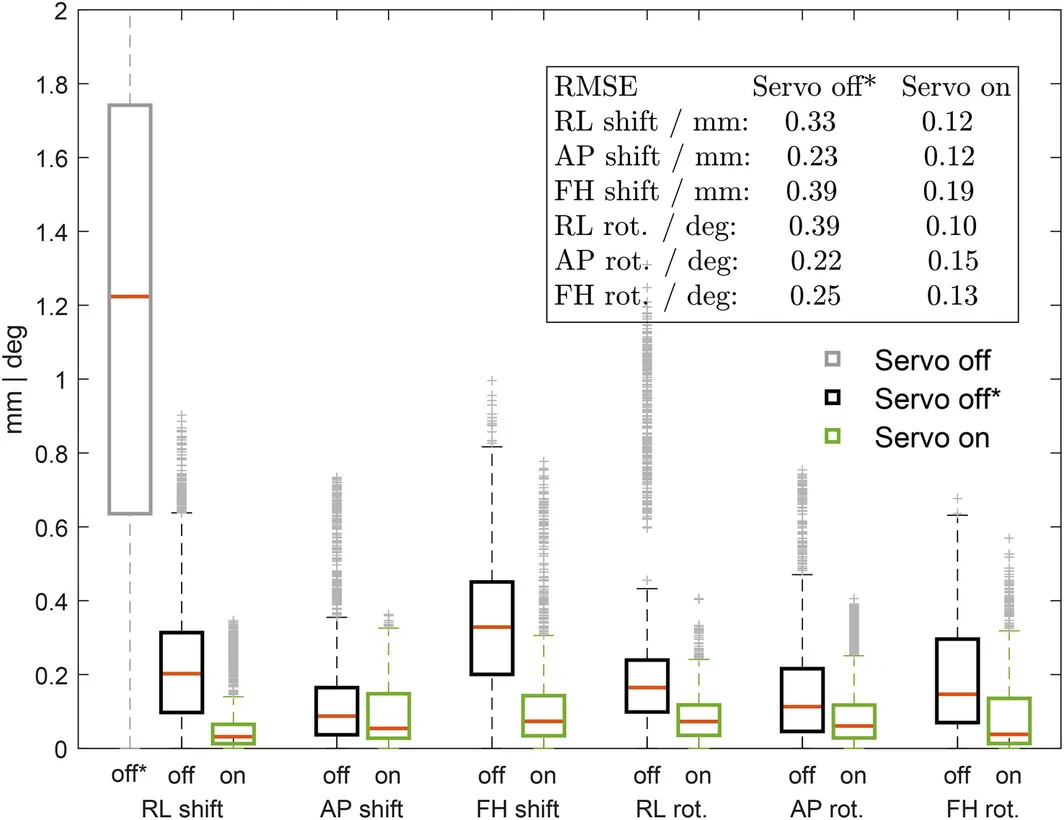
Volume realignment statistics for all in vivo subjects without instructed motion comparing Servo off* (black boxes) and *Servo on* (green boxes). Boxplots for shifts along RL, AP, and FH are shown, as well as the respective rotations. A table of parameter RMSE values is included. Motion is consistently reduced by servo navigation for all parameters. The RL shift (phase encoding direction) includes both subject motion and EPI shifting from frequency drift for *Servo off* (gray box). Frequency drift‐induced RL shifts were retrospectively corrected for Servo off* (black box).

Figure S2 shows the corresponding total motion traces for all subjects. Figure S3 shows that the FD and MD motion levels are comparable for Servo off* and *Servo on*. Servo off* comprises slightly fewer movements, as measured by FD and MD.

### 3.3 Phase Equalization Example

Figure 5 shows phase equalization in the phantom without servo navigation. The mean of the voxel time series is shown for reference. Without phase equalization, the standard deviation (std) is the highest and is strongly reduced by realignment. With phase equalization, the standard deviation is further reduced, and realignment does not give an additional advantage. A frequency drift of about 5Hz/min is captured in the estimated parameters (B), and the zoom (C) shows patterns that repeat with volume acquisition time $T_{vol}$.

**FIGURE 5:**
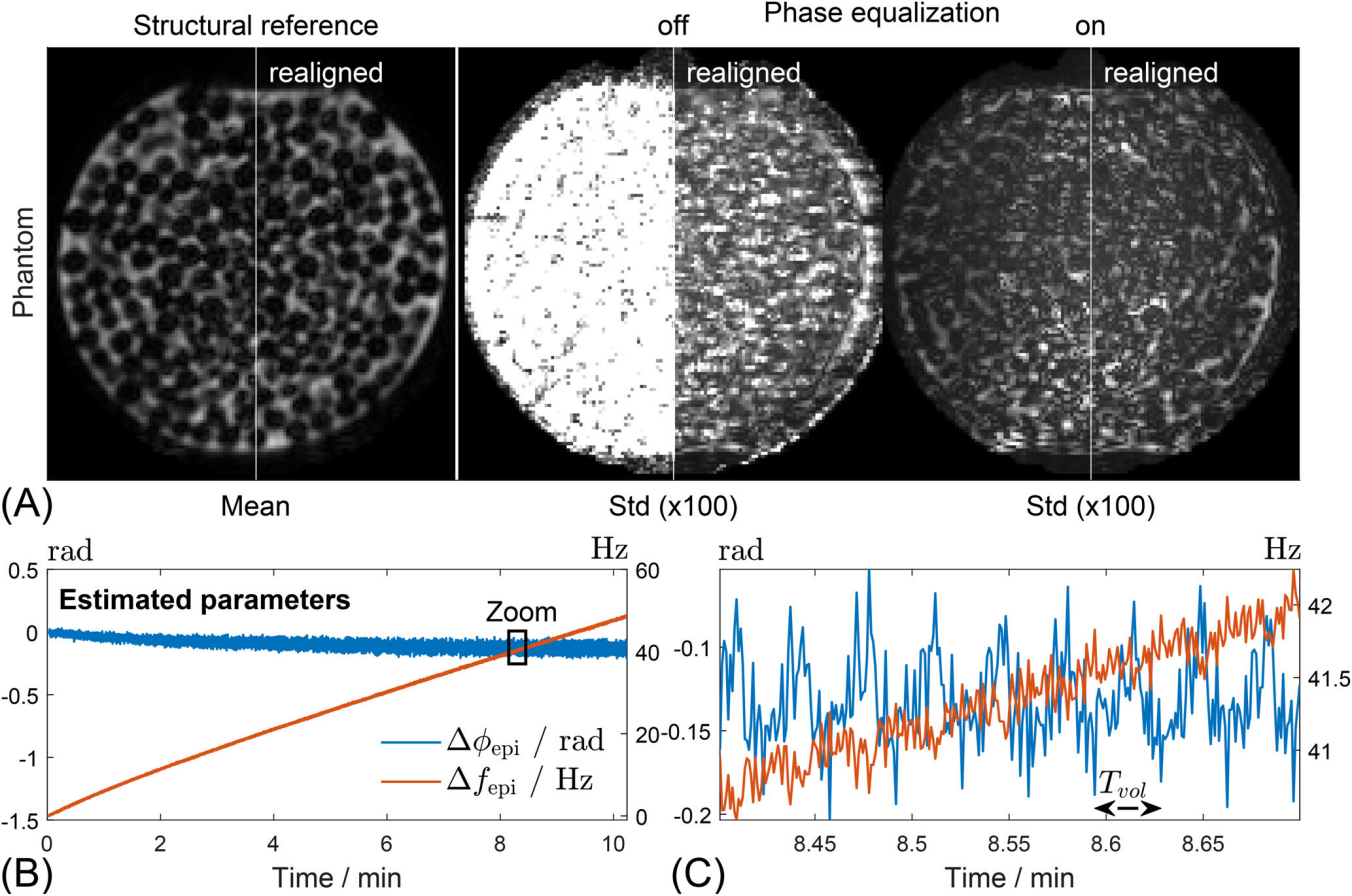
Phase equalization of voxel times series in a phantom scan without servo navigation. (A): Mean and standard deviations (Std) without and with phase equalization. Each image is split by half showing the image before and after realignment. (B): Phase $\Delta \phi_{\text{epi}}$ and frequency $\Delta f_{\text{epi}}=\Delta \omega_{\text{epi}}/(2\pi )$ estimated by phase equalization. (C): Zoom into (B). EPI shifting from the frequency drift is reduced by realignment without phase equalization. Phase equalization captures the frequency drift well (B) and further improves volume consistency by intra‐volume phase correction. Repetitive patterns with $T_{vol}$‐period are visible in (C).

Continuous frequency drift is a major cause of tSNR instability, which appears as EPI shifting in the low‐bandwidth phase encoding direction [56]. Volume realignment is well suited to correct for these inter‐volume shifts, strongly reducing the standard deviation for *phase equalization off*. While realignment can only correct for global transformations between volumes, the phase equalization is capable of addressing both inter‐ and intra‐volume phase and frequency variations including slow frequency drifts and sampling‐related effects. By this, phase equalization reduces the respective artifacts and thus improves volume consistency.

### 3.4 Spectral Parameter Analysis

Figure 6A and B shows the power spectra of the phase equalization parameters compared to the respective navigator spectra. Quadratic detrending of the parameter traces was applied before generating spectra to reduce interference of the spectral contents of frequency drifts and $f_{vol}$‐peaks. The phase equalization parameters contain a Dirac‐comb pattern spaced by $f_{vol}=1/T_{vol}\approx 0.49\;\mathrm{Hz}$, which is associated with the repetitive $T_{vol}$‐spaced patterns of the time domain signals in Figure 5B. Compared to the navigator frequency estimates, the variation in spectral content indicates that the EPI data is affected by transient field effects that differ from those acting on the navigator and are therefore not correctable by the navigator. The reduced noise floor of the phase equalization frequency parameter shows its higher sensitivity.

**FIGURE 6:**
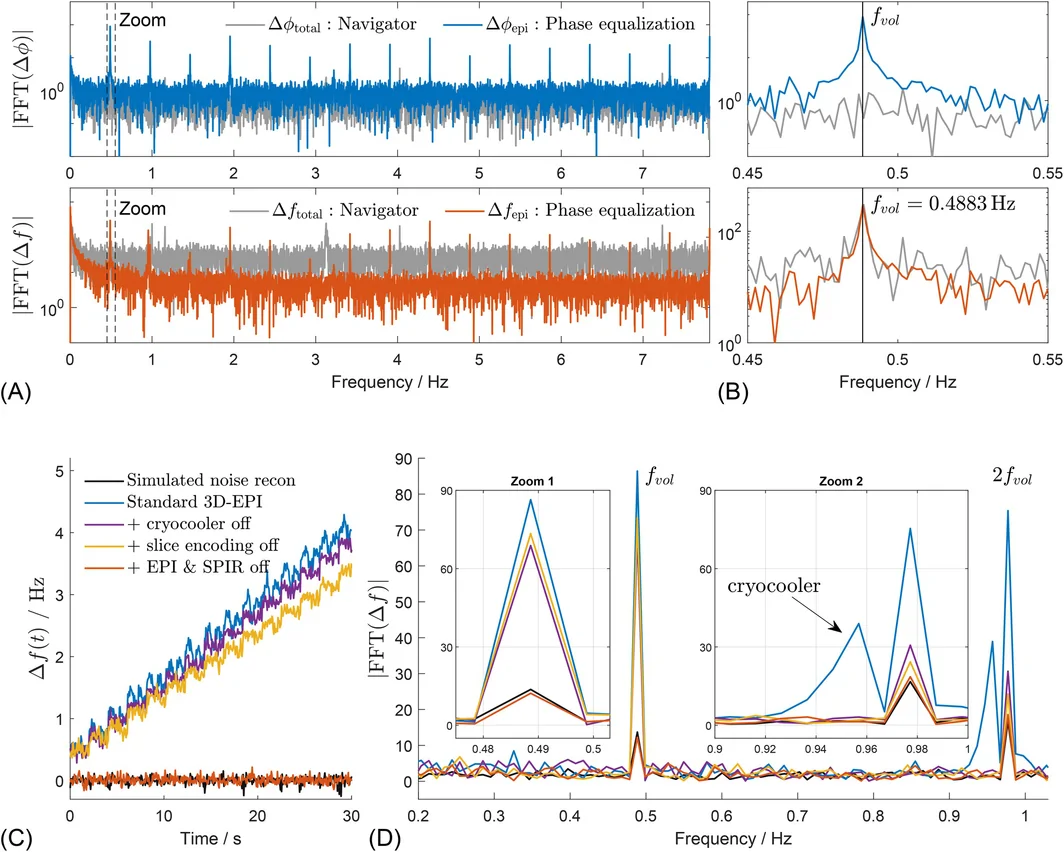
Phase equalization parameter spectra for phantom scans without servo navigation. (A): Power spectra for phase (top) and frequency (bottom) corresponding to Figure 5B. Navigator parameter spectra are shown for reference. (B) Zooms. (C) Frequency time courses estimated by phase equalization for several 30‐sec test scans. (D) Section of power spectra. The spectra in (A) show a $f_{vol}$‐spaced Dirac comb pattern and different spectral contents and noise floors compared to the navigator parameters. $f_{vol}$‐peaks in the frequency spectra of (D) mainly come from gradient characteristics driven by EPI gradients and SPIR fat suppression (red line) as well as field fluctuations at 0.96 Hz from the cryocooler (Zoom 2).

Figure 6C and D shows frequency time courses and spectra estimated by phase equalization for various 30‐sec test scans with fewer volumes. Compared to the standard 3D‐EPI sequence (blue), switching off the cryocooler (purple) reduces the broad peak at 0.96 Hz (Zoom 2). Disabled slice encoding gradients (yellow) barely change the results, whereas switching off all EPI gradients and SPIR fat suppression (red) strongly reduces the $f_{vol}$‐peak (Zoom 1). Hence, phase equalization mainly captures frequency and phase variations at EPI sampling time induced by magnet and gradient imperfections such as drift, vibrations, eddy currents, and the cryocooler. In vivo, phase equalization also captures frequency fluctuations from respiration as shown in Figure S4.

The remainder of the $f_{vol}$‐peaks (red) matches reconstructions with simulated noise (black). In this simulation, the raw k‐space data of each volume was replaced by the data from the first volume with different additive SNR‐matched noise. The simulation illustrates an underlying noise propagation characteristic of the phase equalization algorithm at multiples of $f_{vol}$. This effect is of tolerable amplitude for this data and is probably due to the fact that noisy reference shots are used repeatedly (every $T_{vol}$) for phase estimation, making this effect SNR‐dependent.

### 3.5 In Vivo tSNR Example

Figure 7 compares the tSNR for Subject 6 without instructed motion with and without servo navigation, phase equalization and volume realignment. The worst tSNR performance appears when all options are disabled. By enabling the individual corrections one by one, tSNR improves consistently, showing the best result with all corrections enabled. If both servo navigation and phase equalization are enabled, volume realignment can only slightly improve the results. Note that, *Servo off* and *Servo on* are two separate datasets and could be affected by different underlying motions, while phase equalization, as a pure reconstruction technique, is turned off and on for the exact same raw data.

**FIGURE 7:**
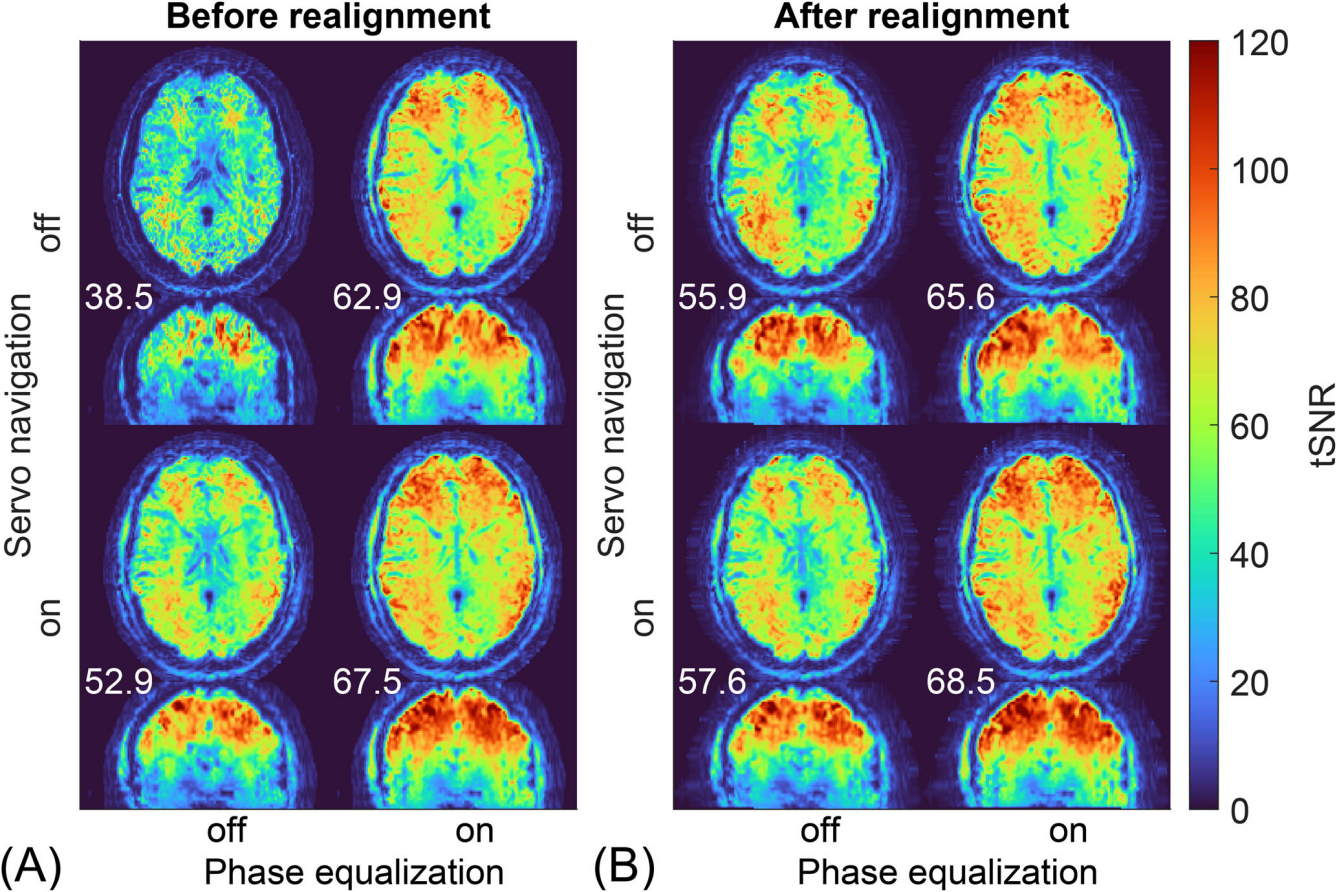
In vivo tSNR example of Subject 6 without instructed motion comparing the performance of servo navigation and phase equalization. (A) Before realignment. (B) After realignment. Mean tSNR values for whole brain are given in the images. Without any of the three corrections (top left) tSNR is lowest mainly due to the frequency drift. Both servo navigation and phase equalization improve tSNR individually, but are most effective together in PEERS. Realignment further contributes a small tSNR improvement.

### 3.6 In Vivo tSNR Statistics

Table 1 shows the mean tSNR of scans without instructed motion over all subjects for different tissue types after realignment including gray matter (GM), WM, cerebrospinal fluid (CSF), and their combination (Whole brain). *Whole brain—not aligned* is given as a reference excluding realignment.

**TABLE 1:** Mean tSNR values of scans without instructed motion over all subjects are given after realignment for different anatomies.

Method	Servo navigation	Phase equalization	GM	WM	CSF	Whole brain	Whole brain ‐ not aligned
Default	Off	Off	56.89	62.55	42.03	56.50	38.82
Servo navigation	On	Off	59.36	63.97	43.08	58.41	56.48
Phase equalization	Off	On	59.96	66.57	44.53	59.96	41.83
PEERS	On	On	63.30	70.06	46.24	63.09	61.97

*Note:* The rightmost column shows *Whole brain* before realignment for reference.

*Abbreviations:* CSF, cerebrospinal fluid; GM, gray matter; WM, white matter.

Both techniques consistently increase tSNR for all anatomies. Without realignment, phase equalization only shows minor improvements in tSNR (8%) compared to 45% with servo navigation. Combining both servo navigation and phase equalization, PEERS achieves an overall whole brain tSNR increase of 60%. The main tSNR degradation factor that is corrected here is the frequency drift. With volume realignment, the improvements in the whole brain are slightly greater for phase equalization (6%) than for servo navigation (3%). Together, an increase in tSNR of 12% is achieved, which is more than adding the individual contributions of servo navigation and phase equalization, supporting the synergistic relationship. Furthermore, the tSNR increase after volume realignment quantifies the positive impact of intra‐volume motion and field corrections, which are not achievable by inter‐volume corrections in the post‐processing. Note that, the relative impact of motion on tSNR depends on the prevalence of motion in the data and these scans were performed without motion instructions and included only healthy, cooperative volunteers.

Beyond the stability metrics targeted by this study, a tentative SPM analysis of the task‐based fMRI data has been performed and is shown in Supporting Information Section S1 and Figures S5 and S6. In this limited data set, PEERS reduced the SPM model residuals and variance related to motion regressors, as might be expected. Interestingly, PEERS also reduced the number of activated voxels. However, interpretation of these observations is limited by missing ground truth, the presence of both false‐negatives and false‐positives, and changes in noise characteristics by PEERS.

### 3.7 PEERS in the Presence of Motion

Figure 8 shows a tSNR comparison for an in vivo case with instructed motion after realignment. With motion, the mean tSNR drops from about 50 (a; reference) to 39 (c). The mean tSNR increases to 42 for phase equalization alone (d) and to 45 for servo navigation alone (e), while reaching 50 for PEERS, which includes both corrections (f). If motion is present, the relative importance of the run‐time motion correction thus increases. Still, the corrected motion case does not achieve the tSNR performance of 63 of the motion‐free case with phase equalization. RMSE values of volume realignment parameters dropped from (0.86mm, 1.1°) without any correction (c) to (0.11mm, 0.2°) for PEERS (f). The in vivo results are supported by phantom data shown in Figure S7, where the phantom was placed on a platform and shifted in and out by 1mm every 10 s. The shifting direction was aligned with the frequency, phase, and slice direction in separate scans.

**FIGURE 8:**
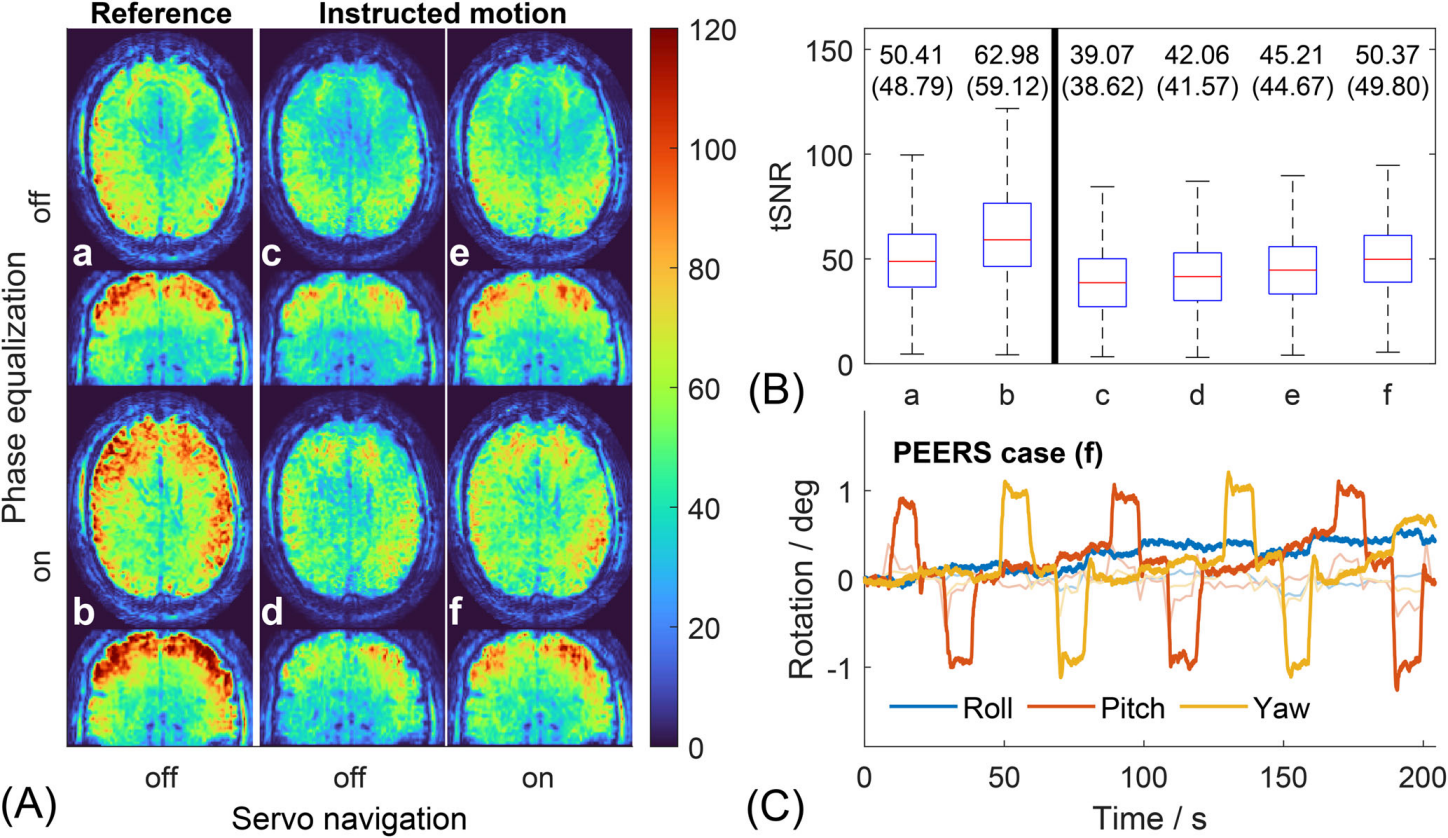
In vivo tSNR example with instructed motion comparing the performance of servo navigation and phase equalization after realignment. (A) tSNR images without (a–b) and with (c–f) motion. (B) Associated tSNR boxplots with mean (median) values. (C) Navigator (bright) and realignment (transparent) rotation parameters for *Servo on*. The boxplots show a clear tSNR reduction by motion without servo navigation and phase equalization (c). With motion, the servo navigation contributes higher tSNR gains than phase equalization, although both consistently improve tSNR.

### 3.8 Frequency Precision Considerations

Figure 9 characterizes the navigator's frequency precision, its impact on tSNR and various ways to mitigate noise propagation into the phase correction including also PEERS. In (A), the standard deviation of the (unfiltered) frequency $\Delta \omega_{total}$ is plotted for different navigator durations $T_{nav}$ for phantom and in vivo scans. The frequency precision grows approximately linearly with the duration of sampling. $1/T_{nav}$‐guidance lines starting from the first 2.3ms‐navigator value are given to visualize the trend. tSNR maps for navigator durations of 3.2 and 11.5ms with and without PEERS are shown in Figure 9B. Phase corrections with unfiltered frequency $\Delta \omega_{total}$ from short navigators result in poor tSNR, which is improved by longer navigators due to increased precision. As shown in (C), phase corrections with filtered frequencies $\Delta \omega_{corr}$ (Equations (3) and (4)) and with relative timing $t_{rel}$ (Equation (5)) considerably improve tSNR also for short 3.2ms‐navigators. Nevertheless, EPI‐based PEERS corrections, which rely on the repetitive sequence structure, outperform navigator corrections in all cases.

**FIGURE 9:**
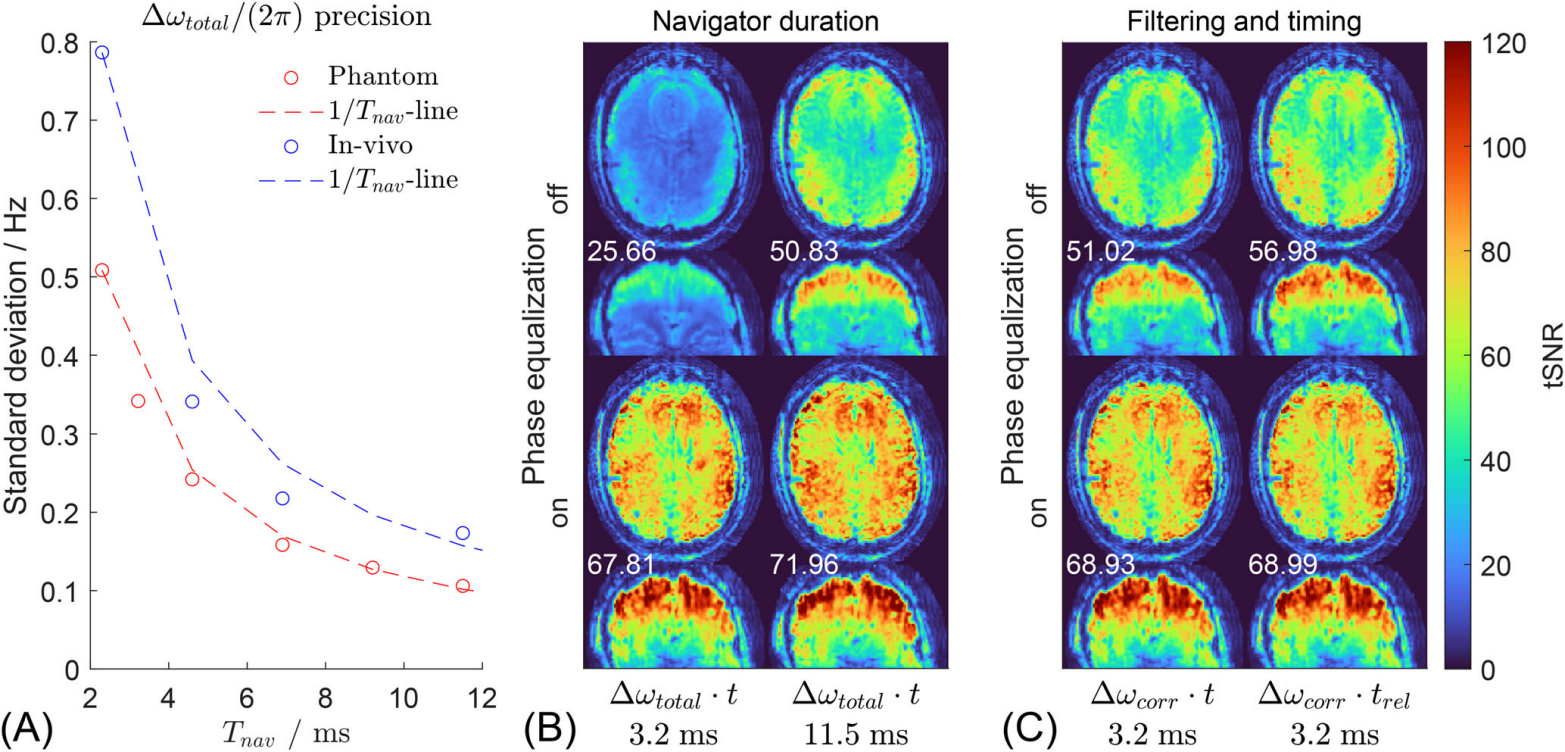
Considerations regarding navigator‐ and EPI‐based frequency estimates for image correction (Subject 4). (A) Frequency precision for different navigator durations $T_{nav}$. (B) tSNR impact of phase equalization and $T_{nav}$. (C) tSNR impact of filtering and relative timing $t_{rel}$. The phase correction type and $T_{nav}$ are indicated below the images. Phase correction is either without ($\Delta \omega_{total}$) or with ($\Delta \omega_{corr}$) filtering and with absolute (t) or relative ($t_{rel}$) timing. Long navigators, filtering and relative timing all improve navigator frequency corrections. EPI‐based phase equalization performs better but requires a repetitive sequence structure.

## 4 Discussion

In this work, servo navigation has been successfully integrated into a segmented 3D EPI sequence for run‐time motion corrected image time series. The method offers on‐the‐fly self‐calibration without additional hardware or extra steps in the workflow. Various methods to reduce noise propagation from the navigator's frequency estimates into the EPI phase corrections have been investigated to enable servo navigation with navigator‐based phase correction. Enabled by run‐time motion correction, PEERS offers robust shot‐wise phase and frequency corrections by exploiting redundancies along EPI time series. Servo navigation mitigates artifacts originating from both intra‐ and inter‐volume motion, and, in combination with PEERS, improves the stability of voxel time series beyond rigid realignment. The stabilizing phase corrections of PEERS enable servo navigation with short 3.2ms‐navigators even for long echo times, underlining the synergistic relationship of PEERS.

### 4.1 Servo Navigation for 3D EPI

In Figures 3 and 4, servo navigation has been shown to capture motion and frequency drifts well, clearly reducing the amount of motion in the imaging raw data. However, residual motion has been observed even with servo navigation in the form of remaining spikes at the onset of instructed pitch rotations and small plateaus while the position was held. The onset spikes are probably caused by the latency of the control system [23]. Our servo navigation implementation currently requires about 300ms to apply an estimated motion state. Small plateaus in pitch‐rotated positions could be caused by secondary motion effects such as pose‐induced coil sensitivity or field changes [18], which can induce parameter biases in servo navigation estimates [44]. Navigator signal variations from coil sensitivity changes should generally be present for both instructed rotations, while only nodding rotations are related to susceptibility‐induced field changes [50]. As the plateaus are mainly seen for nodding, the biases are probably related to pose‐dependent field changes.

It is important to note that the volume realignment, which serves as a reference for motion quantification in this study, is also biased by pose‐dependent contrast variations, such as varying distortions or signal dropouts around the nasal cavities after pose changes. Retrospective volume realignment itself should benefit from run‐time motion correction, because the registered volumes are more consistent, and updating the phase encoding direction in run‐time reduces geometric distortions.

### 4.2 Phase Equalization

Phase equalization has been shown to detect and correct frequency variations from sequence‐related magnet and gradient imperfections in the phantom (Figures 5 and 6) and respiration in vivo (Figure S4). For the sequence used, the main part of the correction is related to frequency changes from magnet drift and gradient imperfections driven by EPI and fat suppression gradients. Using a first‐order phase fit over the echo train, the model can handle only first‐order phase variations over the echo train as caused by long‐term eddy currents. High‐frequency variations, such as those arising from vibrations or short‐term eddy currents, cannot be modeled in this way but are not prominent in the present phase data.

The stability improvements by phase equalization have been reproduced on two different scanners of the same vendor in our lab. As sampling‐related parameter variations are related to general gradient imperfections, the relevance of this correction might depend on the vendor platform and the specific system. However, sequence‐induced drift and gradient imperfections are also expected to be present on other vendor platforms, requiring further validation.

Note that, the phase equalization method has similarities with a self‐navigation approach for 3D multi‐shot EPI published by Moeller et al. [69]. This method also exploits the repetitiveness of a diffusion sequence with multiple diffusion directions and encoding strengths to derive shot‐wise phase navigators. However, in the diffusion case, the phase navigators need to be spatially resolved, resulting in higher computational demands and a more complex phase‐fitting procedure. It is probably more sensitive to instabilities compared to the first‐order phase model presented. Furthermore, the self‐navigation approach by Moeller et al. should also suffer from head rotations, so that an integration with run‐time motion correction could improve its stability, resulting in a PEERS method with a more complex phase model.

### 4.3 Synergistic Relationship

The shot‐wise phase equalization crucially depends on the comparability between the current shot and its peer, that is, the reference shot. This is only given if motion correction is applied in run‐time before the phase equalization, and thus the raw k‐space signal remains unaffected by rotations. Correcting motion retrospectively in the joint multi‐shot k‐space by regridding [79] mixes signals from different shots and, thus, dilutes their phase contributions, which limits the correctability of shot‐specific dynamics by phase equalization. Although phase equalization has been shown to improve tSNR also without servo navigation in vivo (Figure 7) and in the phantom (Figure 5), its effectiveness is reduced and it could potentially even reduce signal stability in the presence of motion.

At the same time, phase equalization alleviates frequency precision requirements on the servo navigation method, because the frequency parameters are fine‐tuned using the EPI data instead. Thus, the combination of both techniques enables intra‐volume frequency corrections for long echo times even with short 3.2 ms‐navigator acquisitions. The navigator‐based frequency and phase correction prior to PEERS is still beneficial, because it keeps the EPI signal phases close to the reference and facilitates the non‐linear phase unwrapping, which requires more cautious treatment in [70]. At the same time, EPI‐based corrections avoid potential phase and frequency mismatches between EPI and navigator sampling, which could arise from their respective transient eddy current and vibration characteristics.

PEERS with servo navigation has been shown to consistently improve tSNR by about 12% in this fMRI study (Table 1), even without instructed motion, with short 2 s volume acquisitions and after volume realignment. Volume realignment still improved tSNR by 1.8% of the maximum tSNR achieved for PEERS. For instructed motion (Figure 8), the tSNR was improved by 16% for servo navigation and by 8% for phase equalization, while together achieving a 29% tSNR increase throughout the brain.

PEERS can also be combined with other prospective motion correction techniques that maintain the signal correspondence in k‐space already during acquisition. In addition, PEERS can also be used to correct scans that involve signal averaging, which is also highly repetitive.

### 4.4 Field Precision Considerations

Navigator frequency precision was found to increase approximately linear with sampling duration (Figure 9A). Longer navigator acquisitions therefore reduce noise in the frequency parameters and stabilize the associated phase corrections at the cost of reduced time efficiency of the navigation. Parameter filtering has also been shown to effectively reduce noise in phase corrections but usually reduces the correction bandwidth at the same time. Relative timing $t_{rel}$ (Equation (5)) performs the corrections without prolonged navigator samplings or reduced correction bandwidth; however, the technique omits bulk phases from frequency variations during the volume sampling. This was found to be effective for short 2 s volume samplings but inappropriate for long volume acquisitions as in gradient echo imaging [44].

In a previous study [44], the servo navigation has been extended to run‐time gradient shimming by calibrating and updating three more gradient shim parameters in the model. Due to the complexity of the frequency corrections studied here, the model has been restricted to zeroth‐order field corrections in this work. First‐ and higher‐order dynamic shimming are subject to future work. Similar to the frequency estimation, uncontrolled noise in the navigator parameter estimates is expected to harm stability for higher‐order corrections at long echo times, and field sensitivity is expected to increase with navigator duration.

### 4.5 Future Research

3D EPI has been shown to offer greater flexibility and sensitivity for high‐resolution fMRI, but is more sensitive to physiological noise and scanner system imperfections due to the segmented volume acquisitions [7]. The combination of servo navigation and phase equalization in PEERS reduces the sensitivity of 3D EPI to physiological and sampling‐related effects. Therefore, the relative comparison of 2D and 3D fMRI is a focus of future projects.

Non‐Cartesian 3D multi‐shot trajectories, such as 3D stack‐of‐spirals [80], have been shown to offer efficient alternatives for volume encoding with potential for higher functional sensitivity. The phase equalization algorithm for arbitrary trajectories has been tested for EPI data, providing results comparable to the EPI‐specific solution. Its performance for non‐EPI trajectories remains to be investigated.

Greater robustness against motion and field fluctuation addresses key weaknesses of 3D EPI and thus holds promise for functional studies using this technique. Stability benefits for fMRI time series have been found at the level of alignment and tSNR. The translation of these benefits to the level of fMRI analysis is a next key question and quite complex [81, 82, 83, 84]. The small data set obtained in this work was not aimed at this task and does not permit any conclusions in this regard. Studies to this end will require substantially greater statistical power and careful choice of representative analysis pipelines.

## Funding

This work was supported by European Union Horizon 2020 Framework Programme (Grant No. 885876).

## Conflicts of Interest

Malte Riedel is a consultant (Malte Riedel Samplewise Elektrotechnik) on MRI topics. Thomas Ulrich is employed by GyroTools LLC, 8044 Zurich, Switzerland. The other authors declare no conflicts of interest.

## Supporting information




**Figure S1:** In vivo example of Subject 1 without instructed motion comparing *Servo off*, Servo off*, and *Servo on*. (Left): Mean and standard deviation (Std) of the voxel time series. (Right): realignment parameters. Servo off* is a reconstruction with volume‐wise frequency correction, where $f_{0}$ is estimated as described for Equation (6). Like for the instructed motion case in Figure 3, the std for *Servo off* shows edge enhancement and choppy patterns. Servo off* mitigates the edge effects from EPI shifting, whereas *Servo on* further reduces variations at the edges and choppy patterns. The realignment parameters are close to zero for *Servo on* showing effective run‐time motion control.
**Figure S2:** Total motion parameters of 10‐min fMRI runs for Servo off* and *Servo on* per subject. For Servo off*, total motion shows volume realignment parameters after retrospective correction of frequency‐induced EPI shifts. For *Servo on*, total motion includes both run‐time motion correction and volume realignment parameters. Framewise displacement (FD) and mean‐absolute displacement (MD) are provided in each plot. Subject 6 shows increased FH shift variances for both methods while showing high tSNR. These variations are correlated to the pronounced respiration patterns including frequency variations of 3 Hz peak‐to‐peak in the navigator $f_{0}$ traces (not shown here) of this subject.
**Figure S3:** Box plots of framewise displacement (FD) and mean‐absolute displacement (MD) over all subjects for Servo off* and *Servo on*. Motion levels are comparable between the two datasets. Servo off* shows slightly lower frame‐to‐frame motion (FD). Absolute motion excursion as measured by mean and median MD is also lower for Servo off*, albeit with an increased standard deviation.
**Figure S4:** Phase equalization parameter spectra for Subject 4 without servo navigation. Power spectra for phase (top) and frequency (bottom) parameters and Zooms (right). Navigator parameter spectra (gray) are shown for reference. The $f_{vol}$‐spaced Dirac comb pattern found in phantom scans is also clearly visible in vivo. Spectral components of the frequency parameter at about 0.25 Hz correspond to respiration and match the navigator estimates well.
**Figure S5:** Overview of EoI and EoM F‐contrasts over all subjects. Thresholding was applied at p<0.001 without family‐wise error correction and spatial smoothing. Default for Subject 6 was dismissed due to missing data.
**Figure S6:** Overview of SPM statistics for task‐based fMRI data. (top) F‐score histograms and SPM model residuals are shown per subject and for all subjects. F‐score thresholding values are indicated by colored vertical lines per subject. (bottom) Table of SPM and motion statistics over all subjects. Default for Subject 6 was dismissed due to missing data.
**Figure S7:** Phantom scans presented as in Figure 8 with intra‐scan shifts in (i) frequency, (ii) phase, and (iii) slice direction. As shifts in phase and (linear) slice encoding direction appear as a phase ramps and constant phase offsets, respectively, phase equalization is capable of compensating these motions well. Shifts in frequency direction (i) as well as rotations (not performed) cannot be represented in the phase equalization model. Hence, tSNR remains low without servo navigation (d).
**Section S1:** SPM analysis of task‐based fMRI data.

## Data Availability

The data that support the findings of this study are available from the corresponding author upon reasonable request.
