# Supplementary material for: Servo Navigation and Phase Equalization Enhanced by Run‐Time Stabilization (PEERS) for 3D EPI Time Series
Source: Magn Reson Med. 2026 Aug 30;96(6):2739–54. doi: 10.1002/mrm.70555 (PMC13619848; doi:10.1002/mrm.70555)
Supplement: Supplementary file 1 [file MRM-96-2739-s001.zip › New folder/supporting_information.pdf]

Supporting Figures

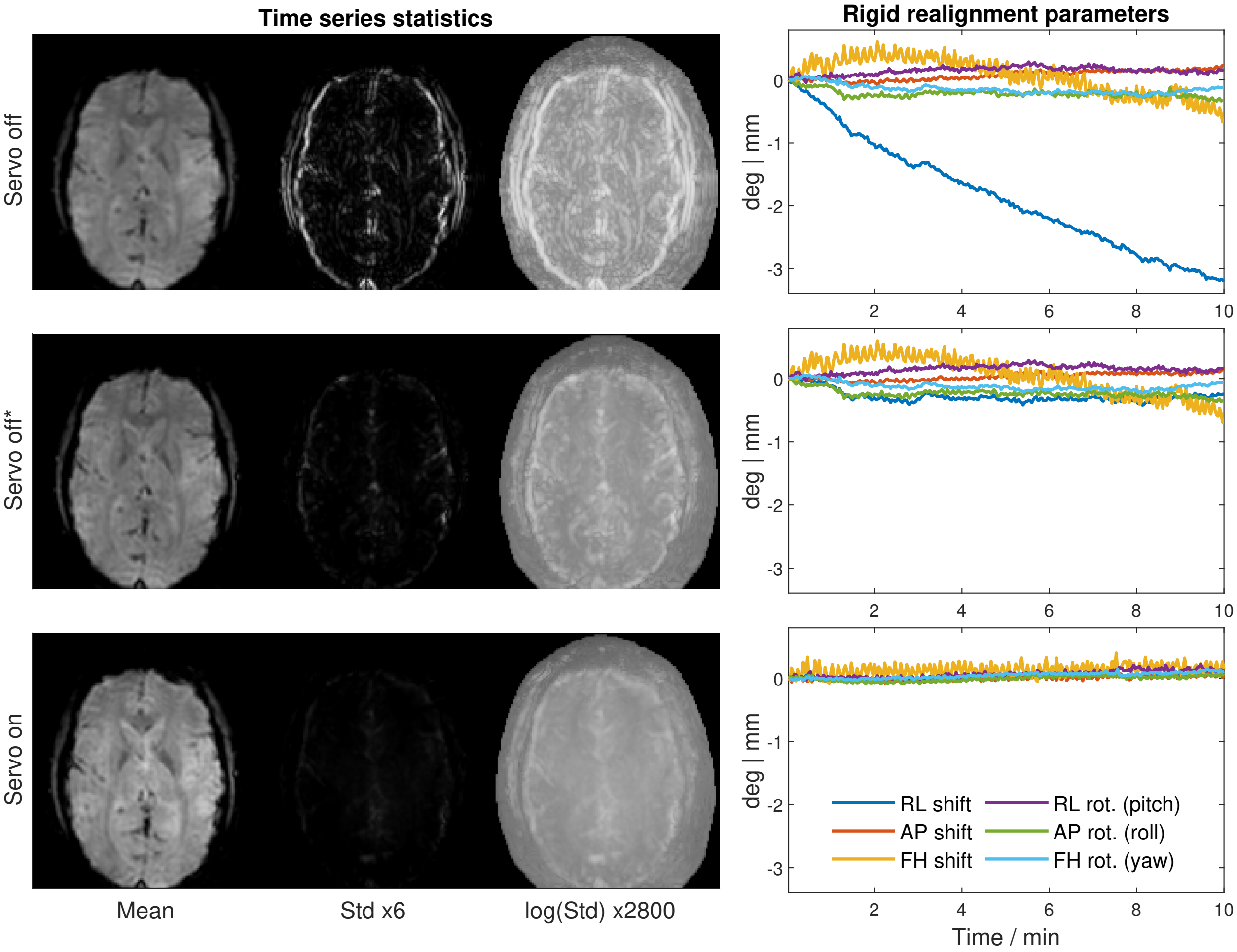

Figure S1: In-vivo example of Subject 1 without instructed motion comparing *Servo off*, *Servo off** and *Servo on*. (Left): Mean and standard deviation (Std) of the voxel time series. (Right): realignment parameters. *Servo off** is a reconstruction with volume-wise frequency correction, where f_0 is estimated as described for Eq. 6. Like for the instructed motion case in Fig. 3, the std for *Servo off* shows edge enhancement and choppy patterns. *Servo off** mitigates the edge effects from EPI shifting, whereas *Servo on* further reduces variations at the edges and choppy patterns. The realignment parameters are close to zero for *Servo on* showing effective run-time motion control.

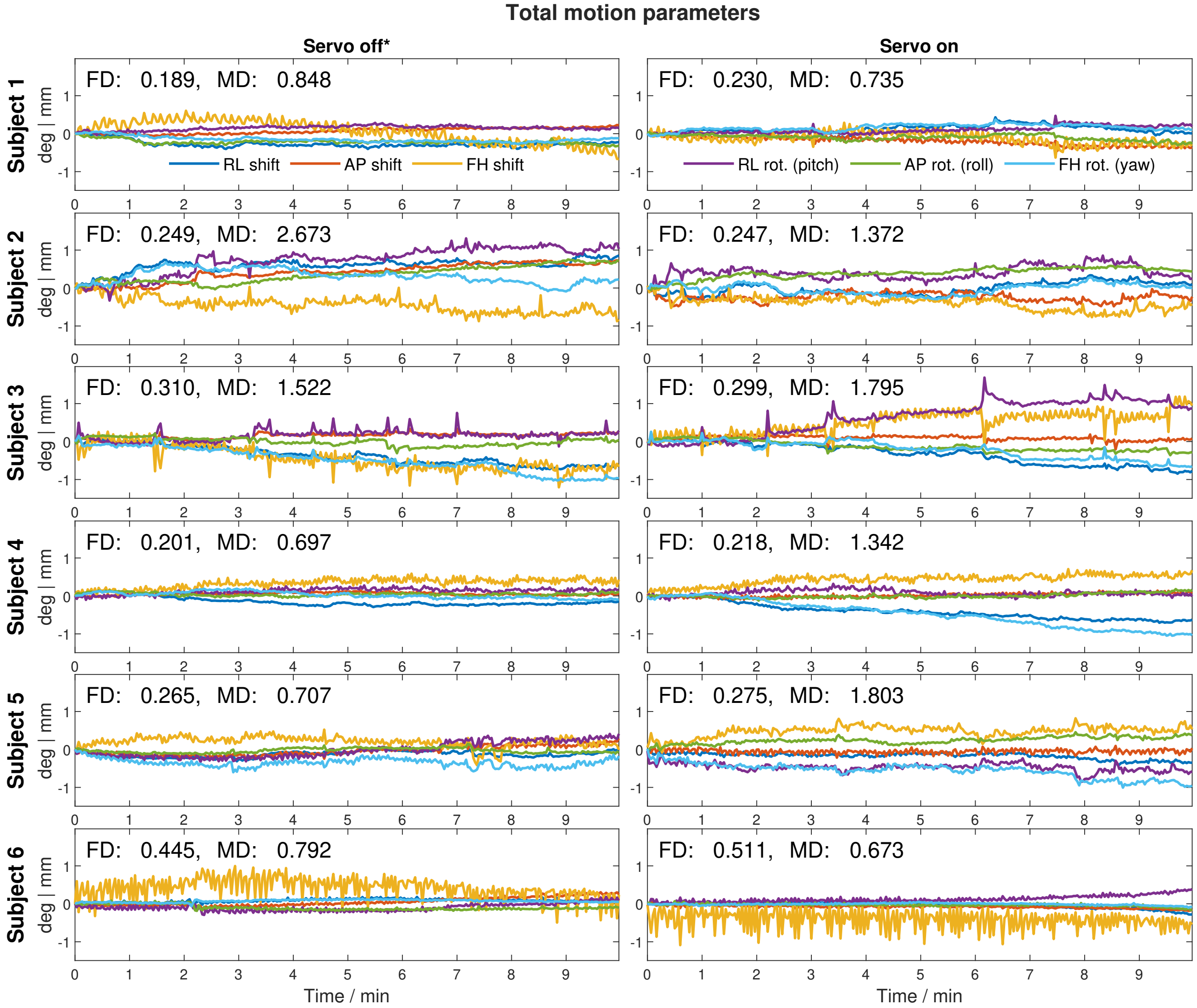

Figure S2: Total motion parameters of 10-min fMRI runs for *Servo off** and *Servo on* per subject. For *Servo off**, total motion shows volume realignment parameters after retrospective correction of frequency-induced EPI shifts. For *Servo on*, total motion includes both run-time motion correction and volume realignment parameters. Framewise displacement (FD) and mean-absolute displacement (MD) are provided in each plot. Subject 6 shows increased FH shift variances for both methods while showing high tSNR. These variations are correlated to the pronounced respiration patterns including frequency variations of 3 Hz peak-to-peak in the navigator f_0 traces (not shown here) of this subject.

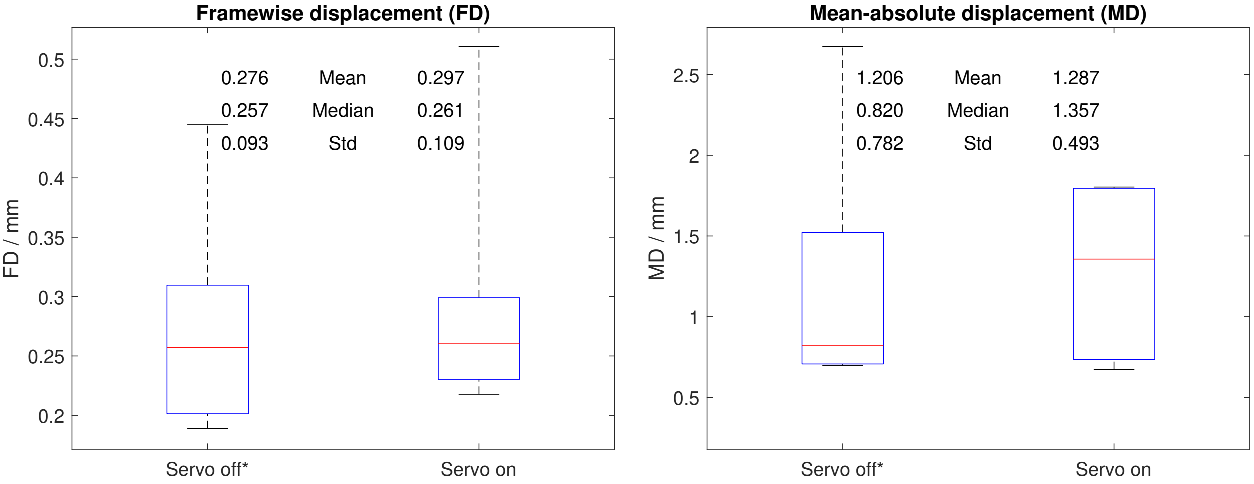

Figure S3: Box plots of framewise displacement (FD) and mean-absolute displacement (MD) over all subjects for *Servo off** and *Servo on*. Motion levels are comparable between the two datasets. *Servo off** shows slightly lower frame-to-frame motion (FD). Absolute motion excursion as measured by mean and median MD is also lower for *Servo off**, albeit with an increased standard deviation.

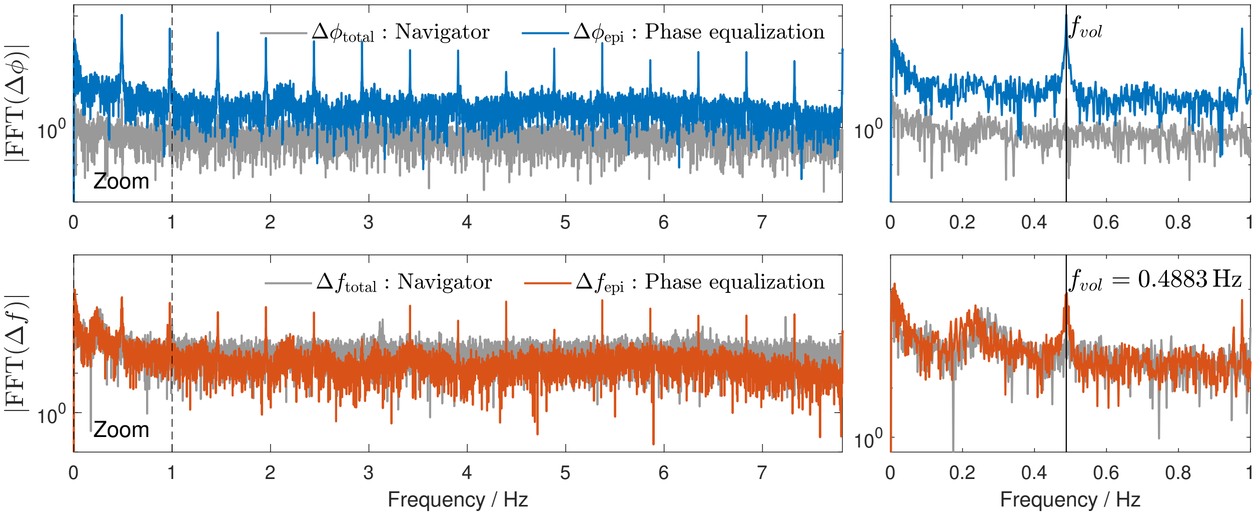

Figure S4: Phase equalization parameter spectra for Subject 4 without servo navigation. Power spectra for phase (top) and frequency (bottom) parameters and Zooms (right). Navigator parameter spectra (gray) are shown for reference. The f_{vol}-spaced Dirac comb pattern found in phantom scans is also clearly visible in-vivo. Spectral components of the frequency parameter at about 0.25 Hz correspond to respiration and match the navigator estimates well.

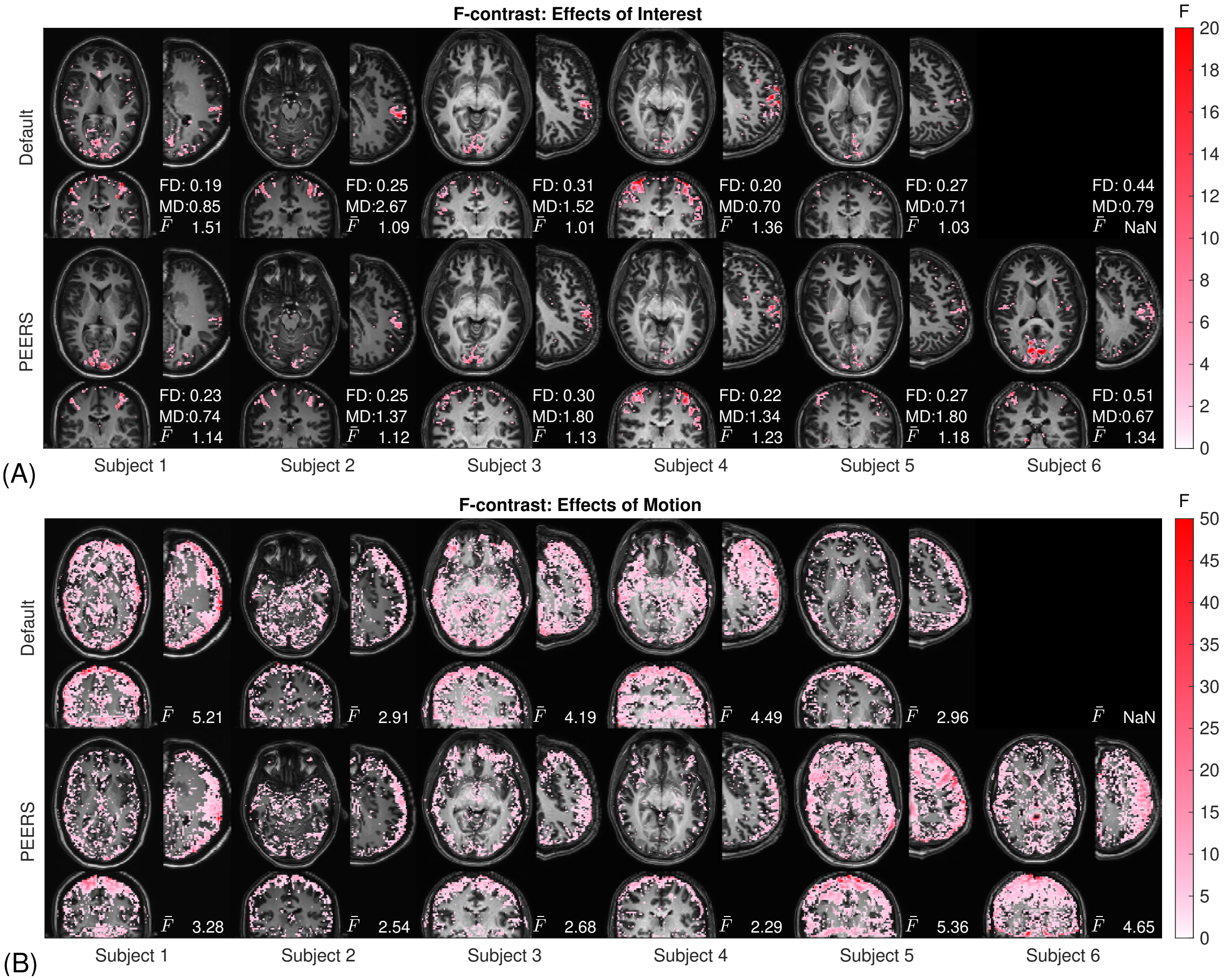

Figure S5: Overview of EoI and EoM F-contrasts over all subjects. Thresholding was applied at p<0.001 without family-wise error correction and spatial smoothing. Default for Subject 6 was dismissed due to missing data.

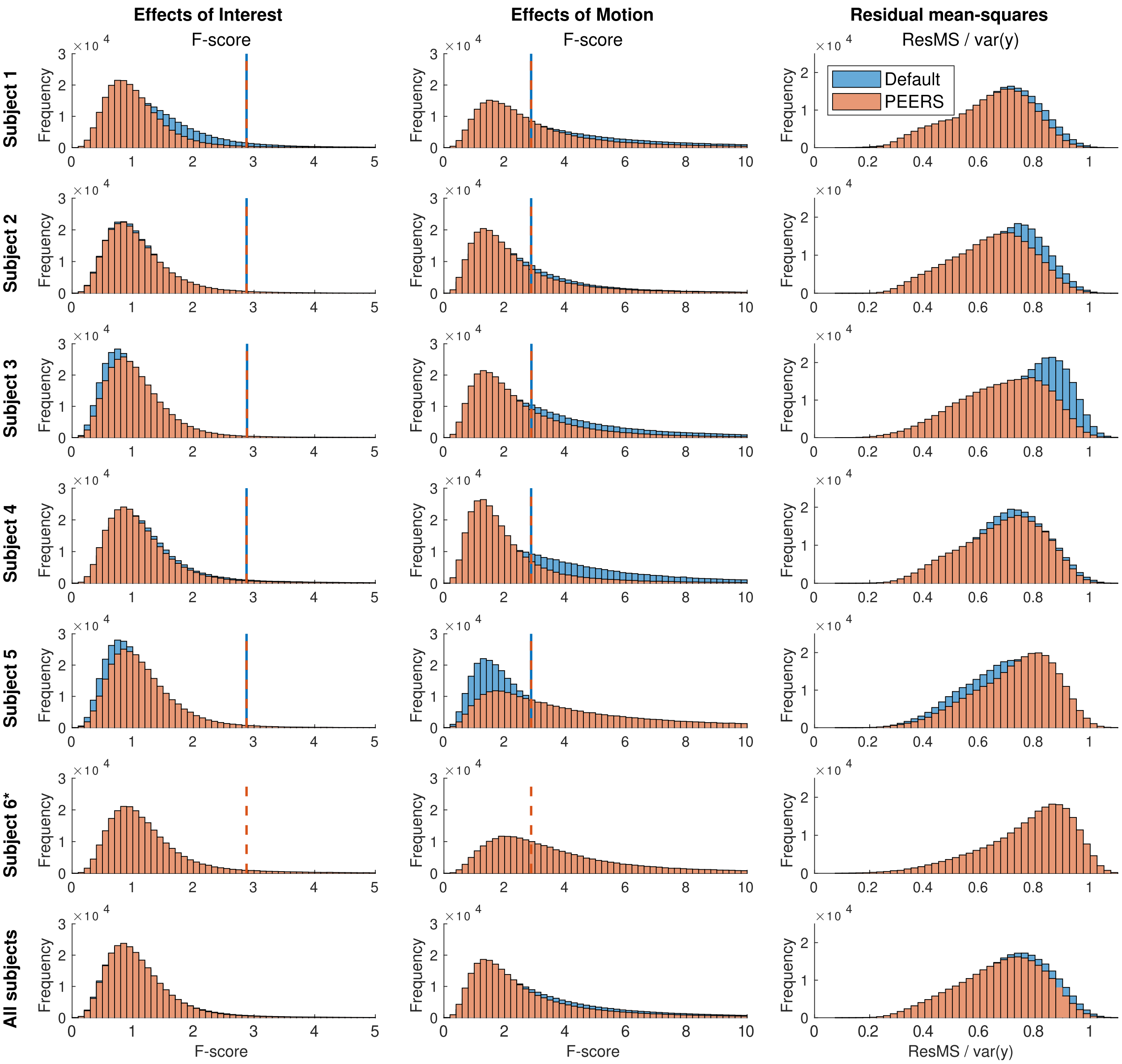

All subjects	Effects of Interest				Effects of Motion				ResMS	Mean motion	
	Mean	Q50%	Q97.5%	#voxels	Mean	Q50%	Q97.5%	#voxels	Norm.	FD	MD
Default	1.19	1.01	3.06	37545	3.92	2.71	14.41	598945	0.707	0.243	1.29
PEERS	1.16	1.01	2.83	30470	3.26	2.18	12.70	457307	0.672	0.254	1.41

Figure S6: Overview of SPM statistics for task-based fMRI data. (top) F-score histograms and SPM model residuals are shown per subject and for all subjects. F-score thresholding values are indicated by colored vertical lines per subject. (bottom) Table of SPM and motion statistics over all subjects. Default for Subject 6 was dismissed due to missing data.

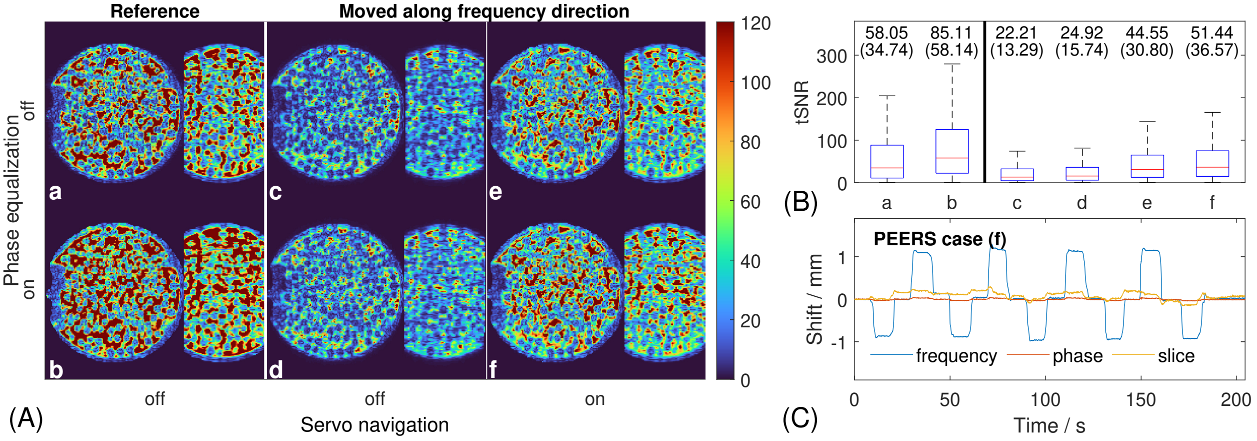

(i) Motion in frequency encoding direction

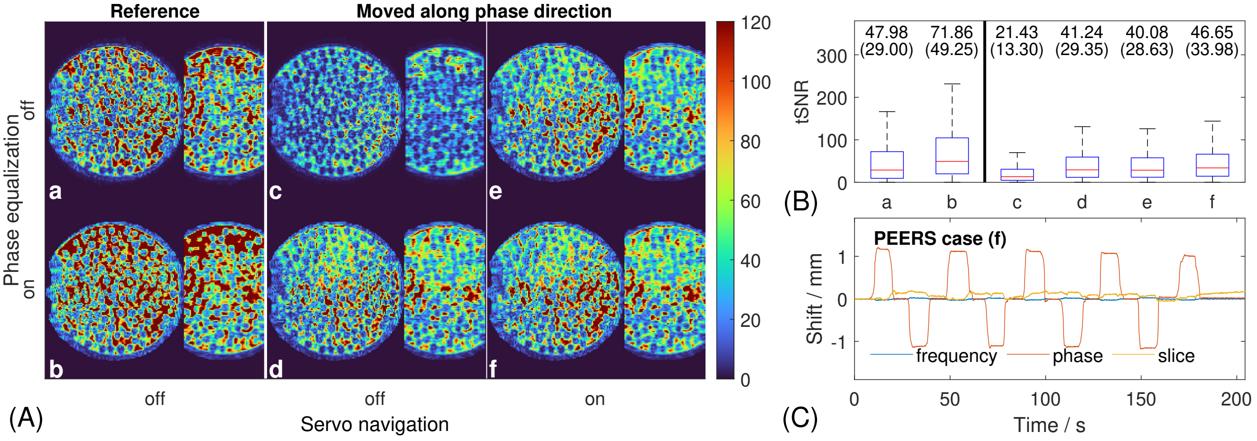

(ii) Motion in phase encoding direction

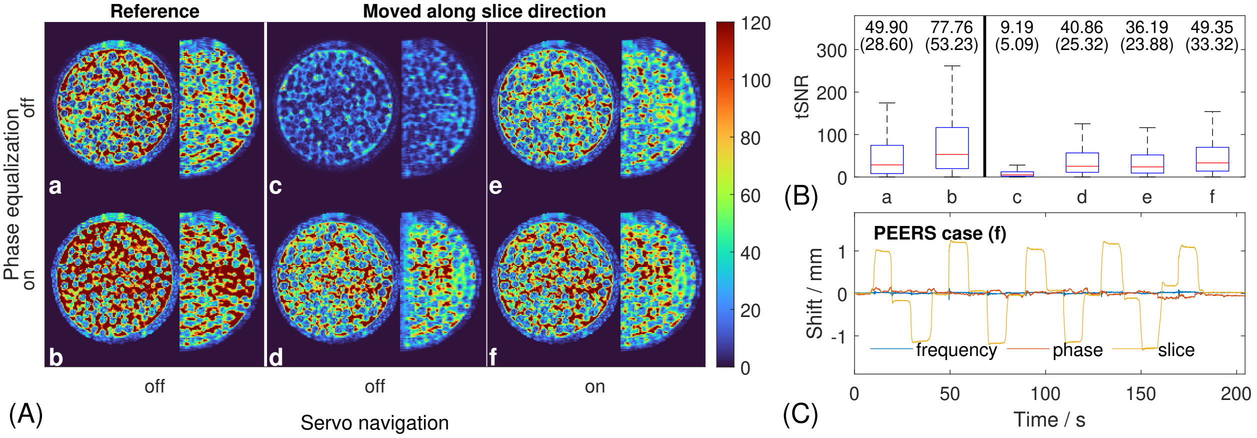

(iii) Motion in slice encoding direction

Figure S7: Phantom scans presented as in Fig. 8 with intra-scan shifts in (i) frequency, (ii) phase and (iii) slice direction. As shifts in phase and (linear) slice encoding direction appear as a phase ramps and constant phase offsets, respectively, phase equalization is capable of compensating these motions well. Shifts in frequency direction (i) as well as rotations (not performed) cannot be represented in the phase equalization model. Hence, tSNR remains low without servo navigation (d).

Supporting Information

Supporting Information 1 - SPM analysis of task-based fMRI data

An SPM analysis was performed for the current task-based fMRI data to evaluate how improvements in tSNR relate to results in functional activity. However, the current study size and study design was deemed insufficient to draw final conclusions about the impact of PEERS for fMRI.

The variance explained by the task-related Effects of Interest (EoI) and the Effects of Motion (EoM) in the form of F-contrasts was compared between the Default (Servo off, no phase equalization) and PEERS. A standard generalized linear model (GLM) was set up in SPM from visual stimuli and button presses. RetroICor regressors, physiological regressors from the respiration belt and pulse oximeter, and 12 motion regressors (6 rigid parameters and 6 derivatives) using the preprocessed total motion traces were included. The GLM comprised standard outlier handling by stick regressors for framewise displacements (FD) >1 mm. Servo off for Subject 6 had to be dismissed, because the physiology file was unfortunately incomplete.

Supporting Figure S5 shows the F-contrasts thresholded at $p<0.001$ (without family-wise error correction). No spatial smoothing was performed to avoid averaging out localized artifacts. The EoI F-contrasts (Supporting Fig. S5A) for Default and PEERS, which are based on raw data from two separate scans (Servo off and Servo on), generally show a high similarity in activation patterns. Framewise displacement (FD) and mean-absolute displacement (MD) are shown in the images along with the mean F-score. Arguably, EoI activation patterns for PEERS have a tendency to be more aligned with the underlying anatomy and appear more consistently in all expected areas (sensorimotor network left right, visual network left right). The F-contrast for Effects of Motion (Supporting Fig. S5B), i.e. the relative variance described by the 12 motion regressors, appears lower for PEERS than for the Default in all subjects except for Subject 5. However, note that Subject 5 comprised three times the absolute motion excursion (MD) for Servo on compared to Servo off (see Supporting Figure S2 for motion traces) and still shows consistent activations in (A) with a higher mean F-score than Default.

Supporting Figure S6 contains the histograms of F-scores and SPM model residuals and a table of statistics. To correct SPM data scaling due to different whitening matrices, model residuals were normalized by the variance of the SPM-preprocessed voxel data y. On average in all subjects, PEERS has similar mean and median EoI F-Scores and lower EoM F-scores as well as reduced

model residuals (Table in Supporting Fig. S6). The 97.5% percentile is reduced by PEERS for both EoI and EoM, resulting in fewer significant voxels. At the same time, motion levels (FD and MD) are comparable and even slightly higher for PEERS.

One could argue that PEERS provides cleaner data, which results in lower SPM model residuals and reduced variance described by motion regressors. However, the F-score picture is complicated. First, the impact of motion on F-scores can change both false positive and false negative rates, potentially pushing F-scores both ways. It is therefore case-dependent and not generally clear whether F-scores and significant voxels should increase or decrease. For these data, the number of significant voxels goes down together with model residuals, which could hint at a reduction of false positives. Second, the F-score is a quotient that contains the estimated residual variance in the denominator. For the Default case, motion residuals are, however, implicitly interpreted as residual variance while corrected in PEERS and noise properties in the reconstructions are found to vary, resulting in quite different whitening matrices. This denominator with different implicit definitions then scales the explained variance before thresholding, which complicates the interpretation of significant activations. Finally, it is even unclear in some aspects how to set up the fMRI pipeline for run-time motion corrected data. Questions like the following arise: Should residual or total motion traces be taken for motion regressors? How are outliers determined for run-time motion correction? How are differences in noise or motion-related pseudo-noise handled in the statistics?

In summary, the picture of F-scores and model residuals is complicated and difficult to compare fairly. This study was designed to investigate how PEERS with servo navigation can mitigate motion corruption and unwanted phase variations in the imaging raw data without and with instructed motion. The improvement of data integrity, such as alignment and tSNR, represents an important technical milestone for future studies in various fMRI applications such as resting state-fMRI, task-based fMRI, connectivity analysis, and high spatial resolution and high temporal resolution applications. The current study size and design was considered insufficient to derive definitive claims for fMRI. The question remains how residual motion in the raw data then correlates with a paradigm and finds its way into functional analyzes despite motion regressors. Dedicated study designs and an in-depth examination of the fMRI pipelines are required to draw well-founded conclusions for fMRI.
